# Biosynthetic pathway of prescription bergenin from *Bergenia purpurascens* and *Ardisia japonica*

**DOI:** 10.3389/fpls.2023.1259347

**Published:** 2024-01-04

**Authors:** Xiang-Yu Liu, Yi-Na Wang, Jiang-Shun Du, Bi-Huan Chen, Kun-Yi Liu, Lei Feng, Gui-Sheng Xiang, Shuang-Yan Zhang, Ying-Chun Lu, Sheng-Chao Yang, Guang-Hui Zhang, Bing Hao

**Affiliations:** ^1^ College of Agronomy and Biotechnology, National and Local Joint Engineering Research Center on Germplasm Innovation & Utilization of Chinese Medicinal Materials in Southwest China, Key Laboratory of Medicinal Plant Biology of Yunnan Province, Yunnan Agricultural University, Kunming, Yunnan, China; ^2^ Yunnan Characteristic Plant Extraction Laboratory, Kunming, Yunnan, China; ^3^ College of Tobacco Science, Yunnan Agricultural University, Kunming, Yunnan, China

**Keywords:** biosynthetic pathway, bergenin, carbon glycosides, C-glycosyltransferase, O-methyltransferases

## Abstract

Bergenin is a typical carbon glycoside and the primary active ingredient in antitussive drugs widely prescribed for central cough inhibition in China. The bergenin extraction industry relies on the medicinal plant species *Bergenia purpurascens* and *Ardisia japonica* as their resources. However, the bergenin biosynthetic pathway in plants remains elusive. In this study, we functionally characterized a shikimate dehydrogenase (SDH), two *O*-methyltransferases (OMTs), and a *C-*glycosyltransferase (CGT) involved in bergenin synthesis through bioinformatics analysis, heterologous expression, and enzymatic characterization. We found that *BpSDH2* catalyzes the two-step dehydrogenation process of shikimic acid to form gallic acid (GA). *BpOMT1* and *AjOMT1* facilitate the methylation reaction at the 4-OH position of GA, resulting in the formation of 4-*O-*methyl gallic acid (4-*O*-Me-GA). *AjCGT1* transfers a glucose moiety to *C-*2 to generate 2-Glucosyl-4-*O*-methyl gallic acid (2-Glucosyl-4-*O*-Me-GA). Bergenin production ultimately occurs in acidic conditions or via dehydration catalyzed by plant dehydratases following a ring-closure reaction. This study for the first time uncovered the biosynthetic pathway of bergenin, paving the way to rational production of bergenin in cell factories via synthetic biology strategies.

## 1 Introduction

Carbon glycosides are a specific type of secondary plant metabolites (Franz and Grun, 1983). Based on the aromatic aglycone structure, carbon glycosides are classified into flavonoid *C-*glycosides, xanthone *C-*glycosides, chromone *C-*glycosides, anthrone *C-*glycosides, and *C-*glycosylated gallic acids (GAs) (Jihen et al., 2017; Wang et al., 2022). Carbon glycosides are formed through the combination of aglycones with sugars catalyzed by *C-*glycosyltransferases (CGTs), which can generate *C-C* bonds to endow acid and glycosidase hydrolysis tolerance (Mori et al., 2021; Bililign et al., 2005; Oualid and Artur, 2012; Chong et al., 2022). Owing to these advantages, *C-*glycoside drugs show remarkably stable drug absorption, molecular recognition, and drug metabolism (Vidal et al., 2013; Singh et al., 2017).

Bergenin is a GA *C-*glycoside that has selective central cough inhibition activity and is the main ingredient of the antitussive drugs Xuedansu Tablet and Capsule widely prescribed in China (Rohit et al., 2017). Bergenin reportedly has anti-inflammatory, anti-anxiety, anti-malaria, anti-cancer, anti-diabetes, anti-hepatotoxicity, immunomodulatory, and neuroprotective pharmacological activities (Rajesh et al., 2011; Liang et al., 2014; Gao et al., 2015; Jitender et al., 2017; Barai et al., 2019; Kumar et al., 2019; Shi et al., 2019; Xiang et al., 2020). Bergenin is widely distributed in higher plants and has been found in more than 90 plant species belonging to 37 genera in 20 families (Mehta et al., 2022). The Chinese Pharmacopoeia lists the roots of *Bergenia purpurascens* and *Ardisia japonica* as natural bergenin resources for the medicinal extraction industry (China Pharmacopoeia Committee, 2020). In both *Bergenia purpurascens* and *Ardisia japonica*, bergenin is distributed the whole plant, but predominantly accumulates and stored in the root (Li et al., 2009).

Despite the significant potential of bergenin, its medicinal value has been greatly compromised due to limited supply. In the last few years, the annual demand for dried bergenin root in the extraction industry has exceeded 2,000 tons (Lv et al., 2017). The excessive and uncontrolled harvesting over several decades has resulted in a significant depletion of natural resources, pushing them towards to exhaustion. Moreover, the cultivation of *B. purpurascens* and *A. japonica* has been challenging due to their preference for high altitudes, mountainous regions, and cold climate conditions. Resulted in an expanded gap in the bergenin raw material market and a gradual increase in prices. Furthermore, the total chemical synthesis of bergenin is insufficient to meet commercial demands (Parkan et al., 2014). Therefore, it is imperative to elucidate the biosynthetic pathways of bergenin and employ synthetic biology approaches for its large-scale production. In recent years, pathway elucidation of *C-*glycosides in plants has received wide interest. Researchers have mainly focused on the discovery and functional characterization of key CGTs involved in *C-*glycoside biosynthesis. At present, more than 50 functional CGTs, mostly involved in *C-*glycosyl flavonoid synthesis, have been identified in plants, including *Oryza sativa*, *Zea mays*, *Fagopyrum esculentum*, *Gentiana triflora*, *Trollius chinensis*, *Glycyrrhiza glabra*, *Scutellaria baicalensis*, and *Dendrobium catenatum* (Brazier-Hicks et al., 2009; Falcone et al., 2013; Nagatomo et al., 2014; Nobuhiro et al., 2015; He et al., 2019; Ren et al., 2020; Wang et al., 2020; Zhang et al., 2020). CGTs can be roughly divided into two functional types: the first type directly adds a glycone to the flavone aglycone to form *C-*glycosyl flavonoids, whereas the second type, which is currently the most reported, binds a sugar moiety to the open-ring form of the 2-hydroxyflavanone skeleton or its monosaccharide and subsequently undergoes cyclization dehydration to form *C-*glycosyl flavonoids (Wang et al., 2020; Chong et al., 2022).

To date, only a few CGTs involved in *C-*glycoside xanthone biosynthesis have been characterized. In *Mangifera indica*, *MiCGT* transfers UDP-glucose to maclurin to generate 3-*C-*glucosylmaclurin, which is subsequently cyclized by xanthone synthase to produce mangiferin (Chen et al., 2015; Chong et al., 2022). In contrast, in *Hypericum perforatum*, the xanthone skeleton is directly *C-*glycosylated to form *C-*glycoside xanthone, and *N4CGT1* and *N4CGT2* directly catalyze the *C-*4 glycosylation of norathyriol to form isomangiferin (Uchida et al., 2021). In *Morus alba* and *Angelica decursiva*, *MaCGT* and *AbCGT*, respectively, are involved in *C-*glycoside coumarin biosynthesis (Chen et al., 2021; Wang et al., 2022).

Bergenin is a *C-*glycosyl derivative of GA that is considered to have the simplest structure among *C-*glycosides that contain a lactone (Taneyama and Yoshida, 1979). Different from the cyclization mechanisms of flavonoid and xanthone *C-*glycosides, the cyclization step in bergenin synthesis occurs on the skeleton and the sugar moiety does not participate in the closure reaction (Franz and Grun, 1983; Chong et al., 2022). Under acidic conditions, the electrophilicity of the oxygen atom in the carboxyl group is enhanced, facilitating its susceptibility to attack by hydrogen atoms in alcohols and leading to the formation of ester intermediates. The protons were supplied by a 1M HCl solution herein. Subsequently, the oxygen atoms of the alcohol undergo electrophilic attacks and form new ester bonds with the carbon atoms in the carboxyl group within the intermediate stage of esterification. In the meantime, the oxygen atom in the original carboxyl group and the carbon atom in the ester intermediate form a new carbonyl group to form lactone. The involvement of the sugar moiety in the formation of the lactone underlies the uniqueness of this class of compounds. For GA *C-*glycosides, the sugar moiety participates in the cyclization reaction, and the lactone is formed through an esterification reaction between the carboxyl group of the skeleton and the *C-*2 hydroxyl group of the sugar moiety (Franz and Grun, 1983).

**Figure 1:**
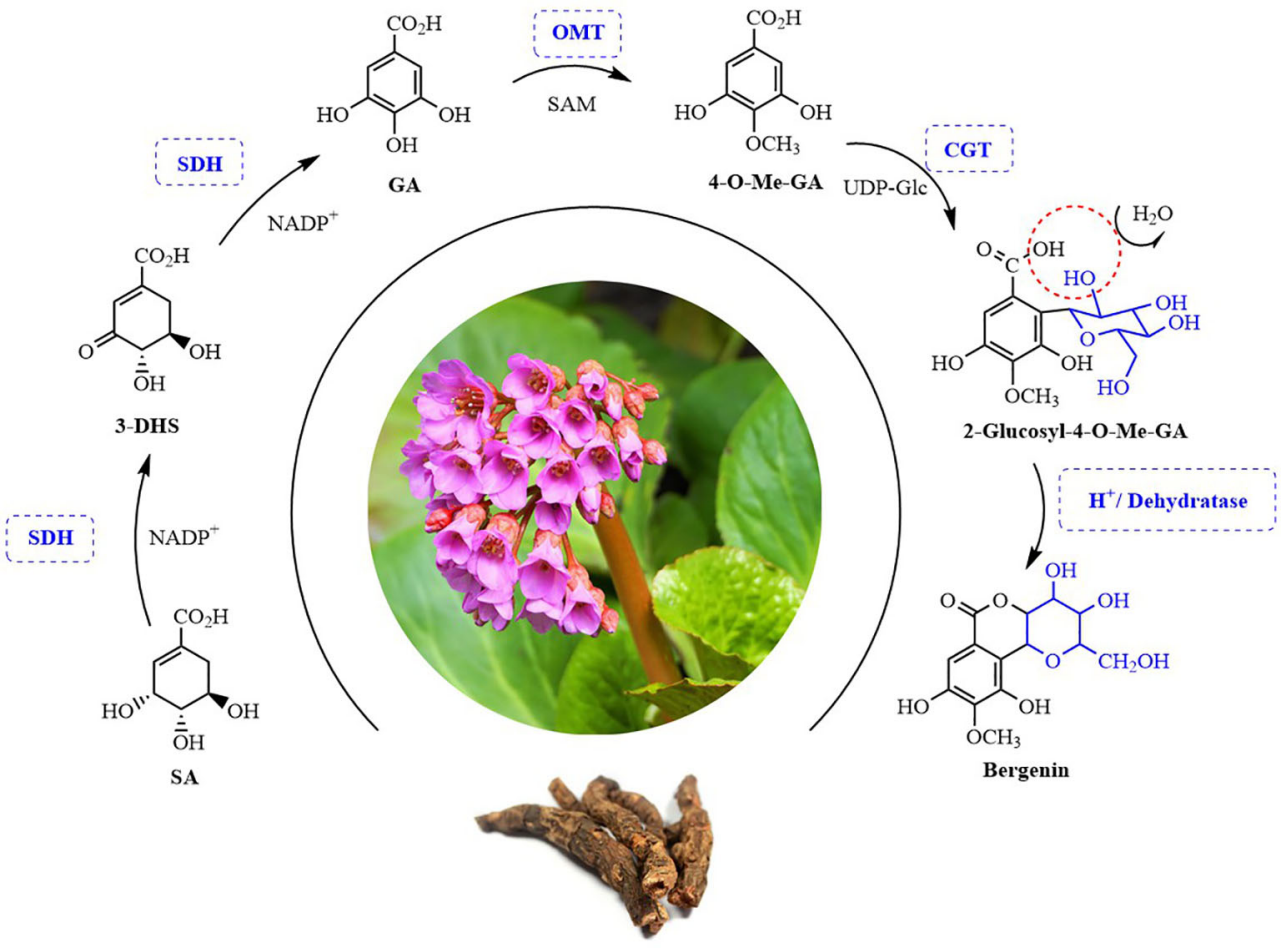
Possible biosynthesis pathway of bergenin in plants. SA, Shikimic acid; 3-DHS, 3-Dehydroshikimic acid; GA, Gallic acid; 4-*O-*Me-GA, 4-*O-*Methyl gallic acid. SDH, Shikimate dehydrogenases; OMT, *O-*methyltransferase; CGT, *C-*glycosyltransferases. SAM, *S*-adenosy-L-methionine; UDP-Glc, Uridine diphosphate-Glucose.

Bergenin is the best-known and major representative of *C-*glycosides. However, its complete biosynthetic pathway remains unclear. In this study, we for the first time elucidated the bergenin biosynthetic pathway. Four candidate genes encoding enzymes involved bergenin biosynthesis in *B. purpurascens* and *A. japonica* were characterized (**Figure 1**). The bergenin biosynthetic pathway starts from shikimic acid (SA), which is catalyzed by *BpSDH2* to produce GA. *BpOMT1* and *AjOMT1* generate 4-*O-*methyl-GA (4-*O*-Me-GA) from GA. The novel *AjCGT1* uses 4-*O*-Me-GA as a substrate to produce 2*-*Glucosyl-4-*O*-Me-GA. After intramolecular dehydration, the closure reaction occurs to form bergenin. The discovery of these enzymes has provided valuable insights into the biosynthetic pathways of bergenin, enabling subsequent efficient *de novo* synthesis of bergenin in cell factories.

## 2 Materials and methods

### 2.1 Plant materials and chemicals

*B. purpurascens* was collected in Lijiang, Yunnan, China and *A. japonica* was collected in Nanning, Guangxi, China. root parts from healthy plants were collected and frozen at –80°C.

SA, 3-DHS, GA, NADP+, 4-*O*-Me-GA, *S*-adenosyl-l-methionine (SAM), UDP-glucose, bergenin, and protocatechuic acid were all purchased from Yuan Ye (Shanghai, China).

### 2.2 RNA extraction, cDNA preparation, and sequencing

The tender plant parts were cut and frozen in liquid nitrogen. Total RNA was extracted using the HiPure HP Plant RNA Mini Kit (Magen, Guangzhou, China) and reverse-transcribed into first-strand cDNA using the PrimeScript™ II 1^st^ Strand cDNA Synthesis Kit (6210A; Takara, Beijing, China).

### 2.3 Transcriptome sequencing and functional annotation

Transcriptome sequencing was performed at Gene Denovo Biotechnology (Guangzhou, China) using the Illumina HiSeq™2000 high-throughput sequencing platform. Raw data were processed by removing reads containing adaptors, reads with a proportion of N >10%, and low-quality reads. The remaining clean reads were subjected to *de novo* assembly using the transcriptome splicing software Trinity, with K-mer set to 25 (Grabherr et al., 2011).

The coding regions (CDS) of the unigenes obtained were predicted using the software tools BLASTX and ESTscan. First, the unigenes were compared to the Nr and Swiss-Prot databases in priority order using BLASTX, with an E-value threshold of 1 × 10^–5^ (Altschul et al., 1997). If a significant match was found in the higher-priority databases, further comparisons with lower-priority databases were omitted. This process resulted in the identification of CDS for the unigenes. The best matching result from the comparison was also utilized to determine the sequence direction of the unigenes. In cases where the unigenes could not be matched in either of the two databases, the CDS were predicted using the ESTscan software. Finally, the predicted CDS were translated into amino acid sequences, and only sequences with a length exceeding 70 amino acids were retained for subsequent in-depth analysis.

### 2.4 Gene sequence analysis and phylogenetic tree construction

Based on searches using the transcriptome data and annotation results from protein databases obtained by local BLAST, key candidate genes encoding shikimate dehydrogenases, *O-*methyltransferase, and *C-*glycosyltransferase were obtained. The open reading frames (ORFs) and amino acid sequences of the SDHs, OMTs, and CGTs were identified using ORFfinder (http://www.ncbi.nlm.nih.gov/gorf/gorf.html). We used InterPro (www.ebi.ac.uk/Tools/InterProScan) to identify functional domains. SDH, OMT, and CGT amino acid sequences from other species were downloaded from the National Biotechnology Information Center database and compared using ClustalW (**Supplementary Table 1**). A maximum likelihood tree was constructed using the IQ-tree software, with 1,000 bootstrap replicates (Nguyen et al., 2015).

### 2.5 Homologous recombination and protein expression

The SnapGene software was used to design specific primers containing homologous arms of the Pet28a vector for the candidate genes (**Supplementary Table 2**). Q5 Mix DNA polymerase (NEB, USA) was used to amplify the genes from cDNA using the following thermal cycling conditions: 98°C for 30 s, 35 cycles of 98°C for 15 s, 58°C for 30 s, and 72°C for 90 s, and finally, 72°C for 10 min. Successful target gene amplification was detected by agarose gel electrophoresis. Successfully amplified genes were recovered from the gel and purified using the EasyPure Quick Gel Extraction Kit (TransGen Biotech, China) and stored at –20°C.

The candidate genes encoding SDHs, OMTs, and CGTs were inserted into the *BamH*I site of pET28a(+) by homologous recombination using NEBuilder^®^ HiFi DNA Assembly Master Mix (E2621) (NEB). The recombinant vector was transformed into *Escherichia coli* BL21 (DE3) cells and sequenced. Individual colonies carrying the correct gene expression vector were inoculated into Luria Broth culture medium supplemented with 50 μg/mL kanamycin and incubated at 37°C under shaking at 220 rpm/min. When the culture reached an optical density at 600 nm of 0.6–0.8, 0.1 mM isopropyl-β-d-1-thiogalactopyranoside was added and the culture was further incubated at 16°C under shaking at 180 rpm/min for 16 h to induce protein expression. Then, the *E. coli* cells were collected by centrifugation at 5,000 rpm and resuspended in binding buffer (50 mM Tris-HCl, 0.2 M NaCl, pH 8.0). The cell membranes were disrupted using an ultrasonic crusher (Scientz-IID, China). The proteins were purified using nickel affinity chromatography. After washing the column with 50 mL of washing buffer (20 mM Tris-HCl, 0.2 M NaCl, and 50 mM imidazole, pH 8.0), the protein was eluted with 10 mL of elution buffer (20 mM Tris-HCl, 0.2 M NaCl, and 250 mM imidazole, pH 8.0). The flow rate was 1 mL/min. Finally, the solution was concentrated using an ultrafiltration centrifuge tube (Merck KGaA, Darmstadt, Germany). Protein purity was confirmed by sodium dodecyl sulfate-polyacrylamide gel electrophoresis, and the protein concentration was determined using a protein quantification assay kit (TransGen Biotech, China).

### 2.6 Functional characterization of SDHs, OMTs, and CGTs

For the functional characterization of SDH proteins, an analytical reaction was carried out in a 100-μL system containing 50 mM Tris-HCl (pH 8.0), 0.1 mM SA, 0.2 mM NADP+, and 20 μg purified enzyme (30°C, 2 h). The reaction was terminated by adding 100 μL of ice-cold HCl (1 M) (Huang et al., 2019). The mixture was centrifuged at 12,000 × *g* for 15 min and the supernatant was analyzed using an Agilent 1290 series ultrahigh-performance liquid chromatography (UHPLC) system (Agilent Technologies, Germany).

For the functional characterization of OMT proteins, an analytical reaction was carried out in a 100-μL system containing 50 mM Tris-HCl (pH 8.0), 0.1 mM GA, 0.1 mM SAM, and 20 μg purified enzyme (35°C, 2 h). The reaction was terminated by adding 100 μL of ice-cold HCl (1 M) (Mageroy et al., 2012). The mixture was centrifuged at 12,000 × *g* for 15 min the supernatant solution is analyzed by UHPLC.

CGT enzyme activity was assessed in a 100-μL reaction system containing 50 mM Tris-HCl (pH 8.0), 0.1 mM 4-*O*-Me-GA, 0.1 mM UDP-glucose, and 20 μg purified enzyme (32°C, 2 h). The reaction was terminated by adding 100 μL of ice-cold HCl (1 M) (Wang et al., 2020). The mixture was centrifuged at 12,000 × *g* for 15 min and the supernatant was analyzed by UHPLC system.

### 2.7 UHPLC analysis

Ten microliters of supernatant were used for UHPLC analysis. The sample was separated on a Waters XBridge Shield RP18 column (4.6 mm × 250 mm, 5 μm). The mobile phase consisted of a 0.1% v/v formic acid aqueous solution (A) and acetonitrile (B). The gradient elution conditions were as follows: 0–8 min, 1%–5% B; 8–13 min, 5%–10% B; 13–20 min, 10%–20% B; 20–25 min, 20%–45% B; 25–35 min, 45%–90% B; 35–40 min, 90% B. The total run time was 40 min. The temperature of the chromatographic column was set to 30°C, and the flow rate was 0.6 mL/min. The detection wavelengths for GA, 4-*O*-Me-GA, and bergenin were 260 nm, 230 nm, and 270 nm, respectively.

### 2.8 Liquid chromatography-tandem mass spectrometry analysis

The reaction products were detected using an Agilent 1290 UPLC Q-TOF UHPLC triple quadrupole MS spectrometer equipped with a heated electrospray ionization source. The MS conditions were as follows: electrospray ionization in negative ion mode, voltage: 3,500 V, fragmentation voltage: 135 V, taper hole voltage: 60V, radio frequency voltage: 700 V, Scanning range: 100–1,000 m/z, scanning mode: SRM. The samples were separated on a Waters XBridge Shield RP18 column (4.6 mm × 250 mm, 5 μm). The mobile phase consisted of a 0.1% v/v formic acid aqueous solution (A) and acetonitrile (B). The gradient elution conditions were as follows: 0–8 min, 1%–5% B; 8–13 min, 5%–10% B; 13–20 min, 10%–20% B; 20–25 min, 20%–45% B; 25–35 min, 45%–90% B; 35–38 min, 90% B; 38–45 min, 90%–100% B. The stripping time was 40 min. The temperature of the chromatographic column was set to 32°C, and the flow rate was 0.6 mL/min. The detection wavelengths of GA, 4-*O*-Me-GA, and bergenin were 260 nm, 230 nm, and 270 nm, respectively.

## 3 Results

### 3.1 *De novo* assembly of transcriptome data and functional annotation of transcripts from *B. purpurascens* and *A. japonica*

mRNA was extracted from the young roots of *B. purpurascens* and *A. japonica*, and cDNA libraries were prepared. The transcriptomes were sequenced and the data were assembled using the Trinity software (**Table 1**). We obtained 309,597,772 and 137,767,838 raw reads in total, and 303,534,544 and 136,379,226 clean reads with a Q20 ratio >97% after quality filtering. The clean data for *B. purpurascens* and *A. japonica* were 44.85 Gb and 20.38 Gb. *De novo* assembly of the clean reads using Trinity software yielded 102,974 and 768,428 unigenes with an N50 length of 1,588 bp and 1,889 bp for *B. purpurascens* and *A. japonica*, respectively. The average unigene length was 881 bp for *B. purpurascens* and 983 bp for *A. japonica*.

**Table 1:** Summary of sequencing and assembly of *B. purpurascens* and *A. japonica*.

	*B. purpurascens*	*A. japonica*
Number of totals Raw reads use in the assembly	309,597,772	137,767,838
Number of totals clean reads use in the assembly	303,534,544	136,379,226
Raw data (Gb)	46.44	20.67
Clean data (Gb)	44.85	20.38
Q20 percentage	98.64%	97.79%
GC percentage	44.08%	44.57%
Number of Unigenes	102,974	768,42
N50 of contigs (bp)	1588	1889
Average length of Unigene (bp)	881	983
Minimum length of Unigene (bp)	201	201
Maximum length of Unigene (bp)	17,394	25097

Functional unigene annotation was based on sequence similarity searches against the Nr (https://ftp.ncbi.nlm.nih.gov/blast/db/FASTA/), SwissProt (https://www.uniprot.org/), KEGG (http://www.kegg.jp/), and KOG (https://www.ncbi.nlm.nih.gov/KOG/) databases. The genes with the highest sequence similarity to the unigene sequences were identified, and functional annotations for the unigenes were obtained based on these results (**Supplementary Table 3**). A total of 44,726 (43.43%) and 37,356 (48.61%) unigenes were thus annotated for *B. purpurascens* and *A. japonica*, respectively.

### 3.2 Phylogenetic analysis of candidate SDHs involved in GA biosynthesis

**Figure 2:**
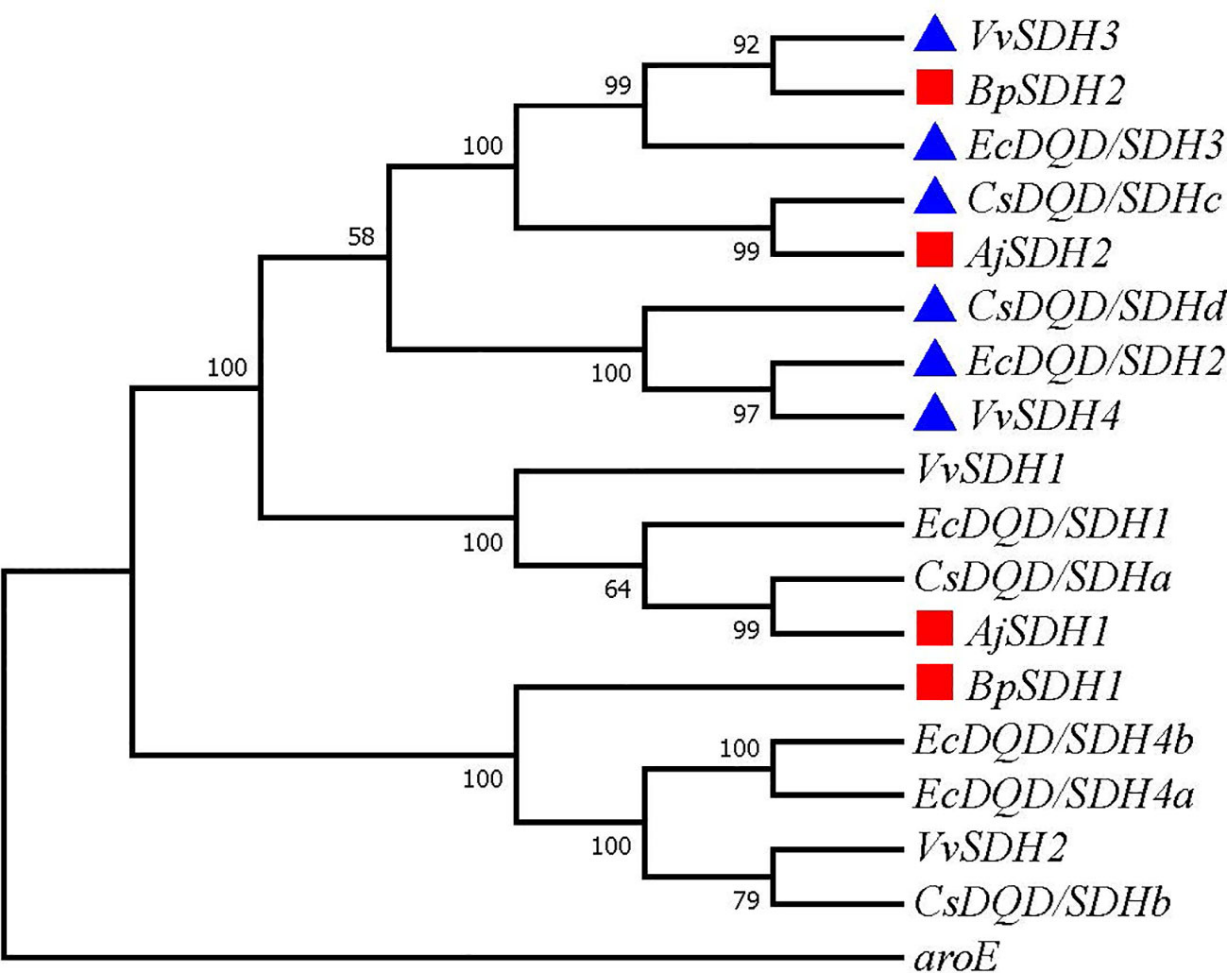
Phylogenetic tree of SDHs involved in GA biosynthesis. A phylogenetic tree was constructed based on the amino acid sequences containing the shikimate dehydrogenases domain from *B*. *purpurascens*, *A*. *japonica*, and other species. The red box represents SDH found in *B*. *purpurascens*, *A*. *japonica*, while the blue box represents reported SDH with GA-producing function. All other species’ SDH sequences were obtained from the NCBI database (**Supplementary Table S3**).

GA is produced from SA by SDH using NADP+ as a hydride acceptor after a two-step dehydrogenation reaction via 3,5-dihydroxydehydro-SA. Subsequently, 3,5-dihydroxybenzoic acid isomerized to form gallic acid (Muir et al., 2011). This pathway has been confirmed in multiple plants (Bontpart et al., 2016; Huang et al., 2019; Tahara et al., 2020). To identify the genes involved in catalyzing the two-step dehydrogenation of SA to produce gallic acid in *B. purpurascens* and *A. japonica*, a phylogenetic tree was constructed using the identified 14 SDHs as references along with 2 SDH proteins from *B. purpurascens* and 2 SDH proteins found in *A. japonica* (**Figure 2**). All sequences information used in the phylogenetic analysis is listed in **Supplementary Table 3**. *BpSDH2* and *AjSDH2* clustered together with *VvSDH3*, *CsDQD/SDHc*, and *EcDQD/SDH3*, and the corresponding proteins showed a 89.83% amino acid sequence similarity (**Supplementary Figure 6**). Since the functionality of SA two-step dehydrogenation to produce GA has been individually confirmed in *Vitis vinifera*, *Camellia sinensis*, and *Eucalyptus camaldulensis* for *VvSDH3*, *CsDQD/SDHc*, and *EcDQD/SDH3*, respectively, we hypothesize that *BpSDH2* and *AjSDH2* might also have similar functions.

### 3.3 Prokaryotic expression and functional characterization of SDHs involved in GA biosynthesis

**Figure 3:**
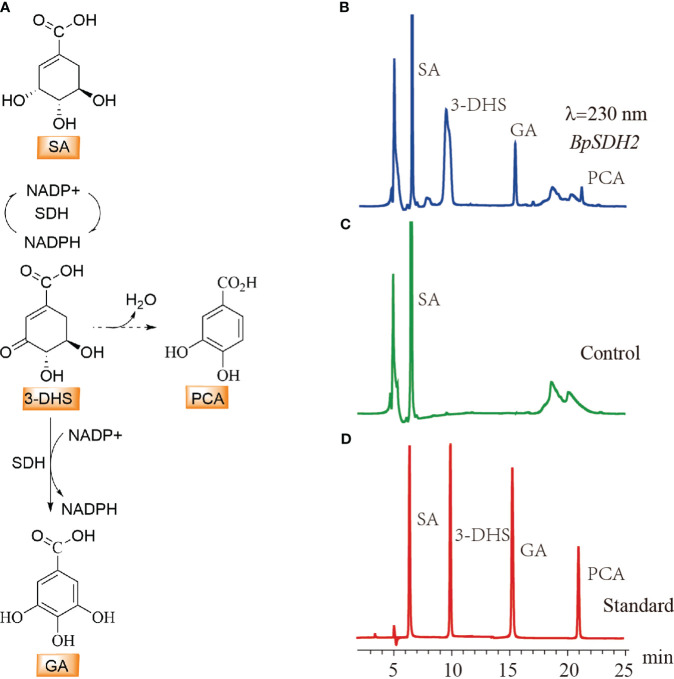
HPLC detection of substrate activity for shikimic acid with BpSDH1 protein. **(A)** The biosynthetic pathway for the conversion of shikimic acid to gallic acid, as well as the possible pathway for the synthesis of protocatechuic acid. **(B)** *In vitro* enzyme activity assay products of shikimic acid with *BpSDH2* and NADP+. **(C)** The reaction of inactivated BpSDH2 protein with shikimic acid and NADP+ serves as a negative control. **(D)** The retention times of SA, 3-DHS, GA, and PCA.

Upon expression in *E. coli*, only *BpSDH2* was obtained in the supernatant and could be used for enzyme assays (**Supplementary Figure 1A**). We first conducted an enzymatic activity assay on the total protein extract of BpSDH2 using SA as the substrate and NADP+ as the hydride acceptor. Two peaks were observed in the chromatogram of the protein extract, whereas there were no corresponding peaks observed in the control (**Figure 3**). The retention times of the peaks matched those of 3-dehydro-SA (3-DHS) and GA standards. The spectrum of BpSDH2 showed LC-MS/MS fragmentation ions of 3-DHS at m/z 171 [M-H]^−^, m/z 127 [M-H-44]^−^, and GA at m/z 169 [M-H]^−^, m/z 125 [M-H-44]^−^, which were consistent with those of the 3-DHS and GA standards (**Supplementary Figures 2**, **3**). These results indicated that the recombinant BpSDH2 protein could catalyze the dehydrogenation of SA in two consecutive steps to produce 3-DHS and GA. HPLC results after the enzymatic assay of *BpSDH2* showed a new characteristic peak that was different from those of 3-DHS and GA (**Figure 3**). GA can also be formed via the synthetic pathway of protocatechuic acid (PCA) or 3,4,5-trihydroxycinnamic acid (Vladimir et al., 2003; Choubey et al., 2015). LC-MS/MS results confirmed that the presence of an unexpected peak with a mass of 153 (m/z, [M-H]^−^) at 21.12 min, which corresponded to the PCA standard (**Supplementary Figures 2**, **3**).

### 3.4 Phylogenetic analysis of candidate OMTs involved in 4-*O*-Me-GA biosynthesis

**Figure 4:**
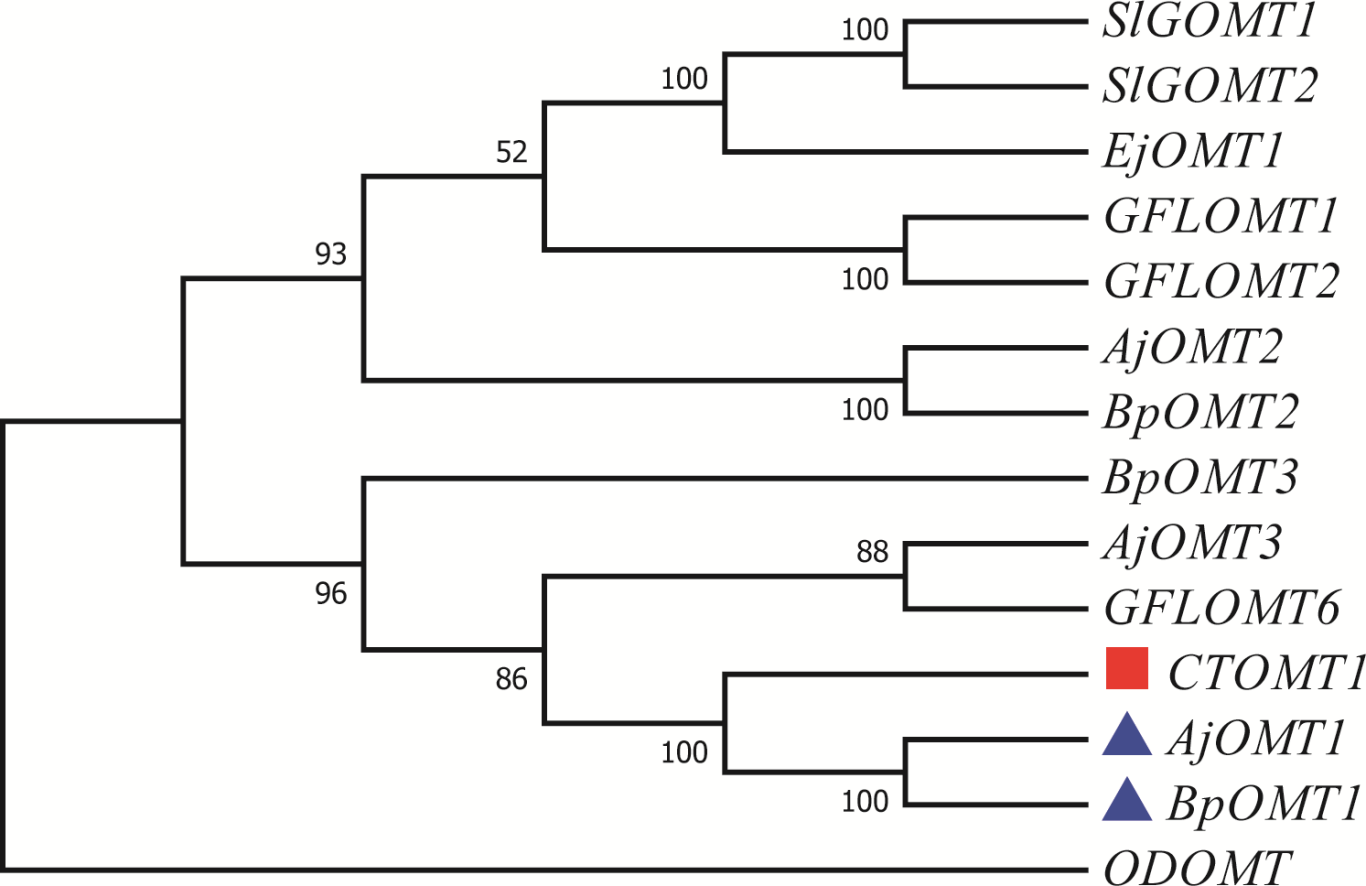
Phylogenetic tree of OMTs involved in 4-*O-*Me-GA biosynthesis. A phylogenetic tree was constructed based on the amino acid sequences containing the *O-*methyltransferase domain from *B*. *purpurascens*, *A*. *japonica*, and other species. The blue boxes represent the candidate OMTs identified from *B*. *purpurascens*, *A*. *japonica*, which are potential enzymes involved in catalyzing the conversion of GA to 4-*O-*Me-GA. The OMTs depicted in red boxes serve as the main reference sequences, as they have been previously reported to have the methylating catalytic function on structures similar to GA. All other species’ OMTs were obtained from the NCBI database (**Supplementary Table S3**).

We assumed that 4-*O*-Me-GA is formed via methylation of GA at the 4-OH position by OMT. While, the phylogenetic tree contained the reported 7 OMTs in plants and OMTs identified in *B. purpurascens* and *A. japonica* was constructed with *ODOMT* from *Oesophagostomum dentatum* (**Figure 4**). *BpOMT1* and *AjOMT1* clustered together with the catechol OMT gene (*CTOMT1*) from *Solanum lycopersicum*, and the corresponding amino acid sequences were highly homologous. *CTOMT1* not only shows catechol OMT catalytic activity, but also methylates other substrates with structures similar to that of catechol, such as protocatechuic aldehyde, pyrogallol, and caffeic acid. Considering the structural similarity between GA and these compounds, we hypothesize that *BpOMT1* and *AjOMT1* catalyze the methylation at 4-OH of GA.

### 3.5 Prokaryotic expression and functional characterization of OMTs involved in 4-*O*-Me-GA biosynthesis

**Figure 5:**
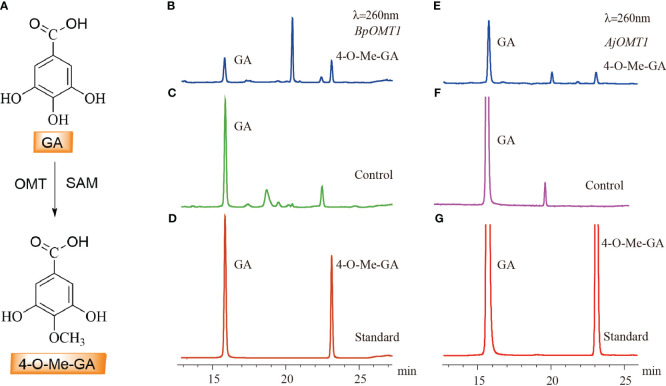
HPLC detection of the *C-*4 methylation activity of OMT protein on GA. **(A)** The biosynthetic pathway of 4-*O-*Me-GA through the *C-*4 methylation of GA. **(B)** The enzymatic activity assay of GA with *BpOMT1* and SAM *in vitro*. **(C)** Inactivated BpOMT1 protein was used as a negative control in the reaction with GA and SAM. **(D)** The retention times of GA and 4-*O-*Me-GA standard. **(E)** The enzymatic activity assay of GA with *AjOMT1* and SAM *in vitro*. **(F)** The reaction of inactivated AjOMT1 protein with GA and SAM serves as a negative control. **(G)** The retention time of authentic standard of GA and 4-*O-*Me-GA.

The candidate genes involved in 4-*O*-Me-GA biosynthesis were heterologously expressed in *E. coli*, and the results showed that both *BpOMT1* and *AjOMT1* were successfully expressed (**Supplementary Figures 1B, C**). The purified proteins were mixed with the substrate GA and the methyl donor SAM and incubated at a constant temperature of 32°C for the enzymatic reaction. HPLC analysis showed a new peak in the enzymatic reaction products of both *BpOMT1* and *AjOMT1* with the same retention time as that of the 4-*O*-Me-GA standard (**Figure 5**). The enzymatic products of *BpOMT1* and *AjOMT1* were confirmed by LC-MS/MS analysis. The spectra of *BpOMT1* and *AjOMT1* products showed LC-MS/MS fragmentation ions of 4-*O*-Me-GA product at m/z 183 [M-H]^−^, m/z 168 [M-H-15]^−^, and m/z 124 [M-H-59]^−^, which were consistent with those of the 4-*O*-Me-GA standard (**Supplementary Figure 4**). These results demonstrated that *BpOMT1* and *AjOMT1* can catalyze the methylation of 4-OH-GA to produce 4-*O*-Me-GA.

### 3.6 Phylogenetic analysis of candidate CGTs involved in bergenin biosynthesis

**Figure 6:**
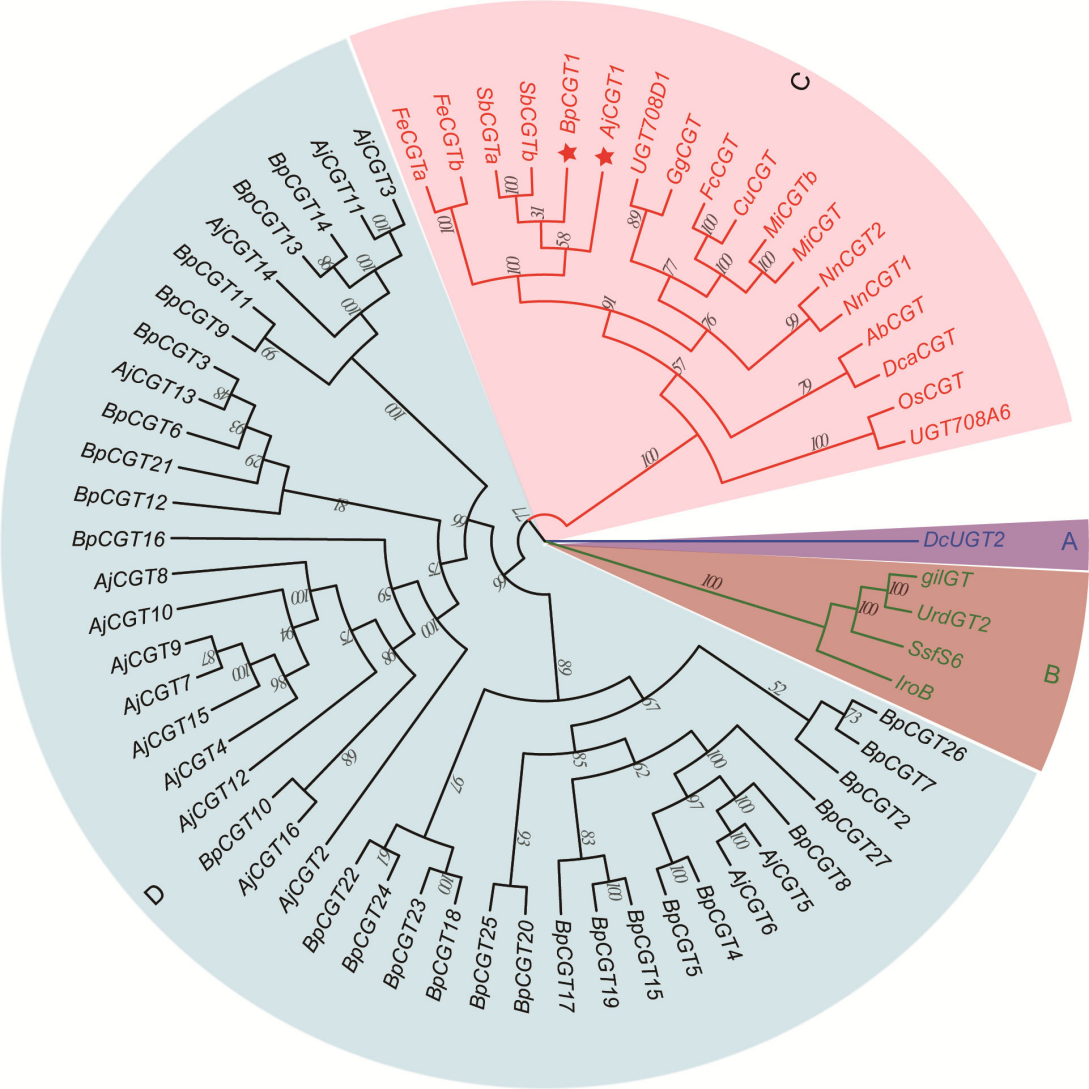
Phylogenetic tree of CGTs involved in bergenin biosynthesis. The sector **(A)** highlights the CGT sequences of glycosyltransferases found in animals, while sector **(B)** highlights those found in bacteria. Sector **(C)** represents the reported CGTs with glycosyltransferase function in plants. Sector **(D)** represents the UGTs identified in *B*. *purpurascens* and *A*. *japonica* by transcriptomics. The red pentagrams represent the candidate CGTs identified in *B*. *purpurascens and A*. *japonica*. The CGTs of other species in sectors A, B, and C were obtained from the NCBI database (**Supplementary Table S3**).

We assumed that 4-*O*-Me-GA is glycosylated with UDP-glucose at the *C-*2 position by CGT, and a lactone ring is formed to produce bergenin after intramolecular dehydration catalyzed by a dehydratase. Based on the conserved motif of UDP-glycosyltransferases (UGTs; PSPG BOX), 43 UGTs were screened from the transcriptome data of *B. purpurascens* and *A. japonica*. A phylogenetic tree was constructed with the 43 screened and characterized CGTs (**Figure 6**).

The genes were clearly divided into four groups, including animal, bacterial, and plant *UGTs* and those of *B. purpurascens* and *A. japonica*. Among all UGTs identified in *B. purpurascens* and *A. japonica*, only *BpCGT1* and *AjCGT1* clustered together with the reported plant CGTs in the phylogenetic tree. The corresponding amino acid sequences displayed a high degree of similarity to these plant CGTs. Based on these results, it can be inferred that *BpCGT1* and *AjCGT1* may possess CGT activity to catalyze the substrate 4-*O*-Me-GA to produce bergenin.

### 3.7 Prokaryotic expression and functional characterization of CGTs involved in bergenin biosynthesis

**Figure 7:**
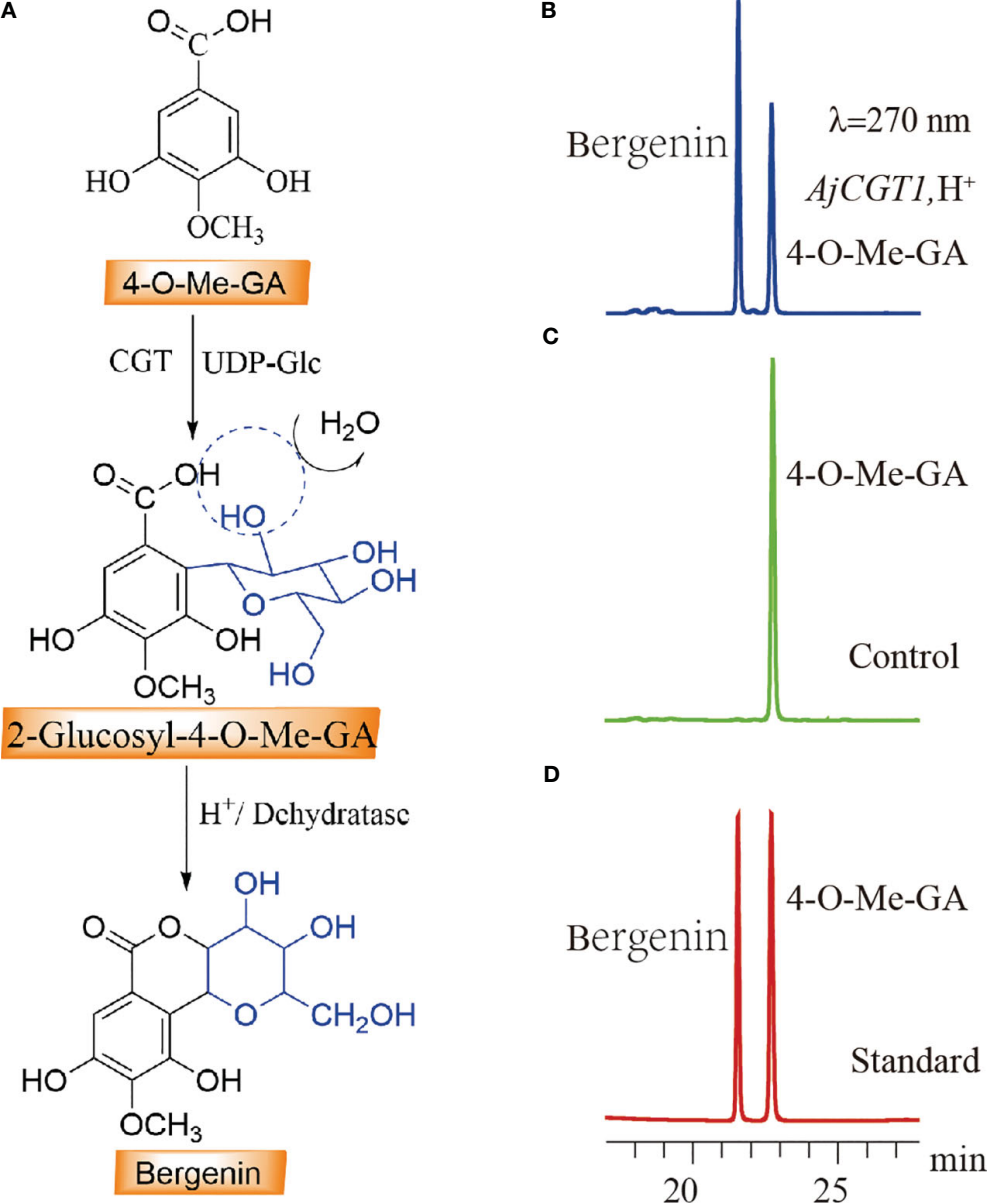
HPLC detection of the *C-*2 glycosylation activity of CGT protein on 4-*O-*Me-GA. **(A)** The biosynthetic pathway of bergenin through the *C-*2 glycosylation of 4-*O-*Me-GA. **(B)** The enzymatic activity assay of 4-*O-*Me-GA with *AjCGT1* and UDP-Glc *in vitro*. **(C)** Inactivated AjCGT1 protein was used as a negative control in the reaction with 4-*O-*Me-GA and UDP-Glc. **(D)** The retention times of the standard compounds 4-*O-*Me-GA and bergenin.

Both *BpCGT1* and *AjCGT1* were expressed in *E. coli* to assess their catalytic activity. Both *BpCGT1* and *AjCGT1* were successfully expressed and purified (**Supplementary Figures 1D, E**). The purified proteins were incubated with 4-*O*-Me-GA and UDP-glucose, and the products were treated with H^+^ before HPLC analysis. Bergenin production was not detected in the *BpCGT1* assay, whereas for *AjCGT1*, a new peak appeared with the same retention time as the bergenin standard (**Figure 7**). The spectrum of *AjCGT1* products showed LC-MS/MS fragmentation ions of bergenin at m/z 327 [M-H]^−^, m/z 312 [M-H-15]^−^, m/z 249 [M-H-78]^−^, and m/z 234 [M-H-93]^−^, which were consistent with those of the bergenin standard (**Supplementary Figure 5**). This result indicated that *AjCGT1* can catalyze the glycosylation of 4-*O*-Me-GA at the *C-*2 position, which is followed by cyclization by a dehydratase or under acidic conditions to form bergenin.

## 4 Discussion

Our findings corroborate that GA serves as the primary substrate for bergenin biosynthesis and plays a crucial role in the production of various secondary metabolites in plants (Franz and Grun, 1983; Petra et al., 2001). GA has been extensively studied for more than half a century (Dewick and Haslam, 1969). Isotope labeling experiments have revealed that it is primarily formed through direct dehydrogenation of SA (Ishikura et al., 1984). Crude extracts of birch leaves have been found to undergo a reaction with 3-DHS and NADP+, resulting in the production of GA. This finding provides further evidence that the intermediate 3-DHS in the shikimate pathway serves as a precursor for GA biosynthesis in plants (Vladimir et al., 2003). SDH is a multifunctional enzyme that also catalyzes the reversible reduction of 3-dehydroshikimate to shikimate (Muir et al., 2011), thereby playing a crucial role in the biosynthesis of aromatic compounds. Recent studies have validated these findings for *VvSDH3* and *VvSDH4* in grapes, *CsDQD/SDHc* and *CsDQD/SDHd* in tea plants, and *EcDQD/SDH2* and *EcDQD/SDH3* in *Eucalyptus camaldulensis* (Bontpart et al., 2016; Huang et al., 2019; Tahara et al., 2020). In this study, we discovered that *BpSDH1* is capable of catalyzing two consecutive dehydrogenation reactions on SA to produce 3-dehydro-SA and GA, providing further evidence for the derivation of GA via the SA pathway in plants (Werner et al., 1997; Roland et al., 2004). In addition, we observed an anomalous phenomenon in the reaction products, wherein PCA was detected alongside 3-DHS and GA. PCA is primarily generated through the dehydration of 3-DHS, a process that has been extensively researched in microorganisms (Elsemore and Ornston, 1995; Muir et al., 2011; Brückner et al., 2018; Kim et al., 2020). However, it remains to be verified whether PCA in this reaction is formed through the dehydration of 3-DHS or other mechanisms.

OMTs play a crucial role in plants by catalyzing the transfer of a methyl group from SAM to hydroxyl groups on receptor molecules (Ibrahim et al., 1998; Joseph et al., 2003; David, 2005). They are actively involved in the biosynthesis of diverse polyphenolic compounds. Although numerous OMTs involved in the methylation of polyphenols have been identified, such as *SlGOMT1* in *Silene latifolia* and *EjOMT1* in *Eriobotrya japonica*, which catalyze the formation of veratrole from guaiacol (Gupta et al., 2012; Takao et al., 2016). Guaiacol is produced through the methylation of catechol by *CTOMT1* in tomato (Mageroy et al., 2012). *SaOMT2* in *Sorbus aucuparia* has exhibited some activity towards caffeic acid and 5-hydroxyferulic acid (Khalil et al., 2015); however, no enzyme capable of methylating GA had been identified to date. In this study, we discovered that *BpOMT1* and *AjOMT1* both exhibit *O-*methyltransferase activity towards the 4-OH position of GA, resulting in the formation of 4-*O*-Me-GA upon the addition of SAM as a methyl donor. Moreover, *CTOMT1* and *SaOMT2* exhibit substrate promiscuity towards compounds that share structural similarity with GA, which is in line with the homology results from the phylogenetic tree analysis (Mageroy et al., 2012; Khalil et al., 2015).

Since the first experimental validation of a CGT (OsCGT, *UGT72B1*) in *Oryza sativa* ssp. indica, numerous key CGTs involved in the synthesis of *C-*glycosyl flavonoids, xanthone *C-*glycosides, and coumarin *C-*glycosides have been verified in plants (Brazier-Hicks et al., 2009; Wang et al., 2020; Chen et al., 2021; Uchida et al., 2021). The formation process of these *C-*glycosides can be roughly divided into two steps. First, a CGT catalyzes the *C-*glycosylation reaction on the open-ring conformation of 2-hydroxyflavanone or benzophenones (Negi et al., 2013; Chen et al., 2015; Feng et al., 2021; Sun et al., 2022). Subsequently, the products undergo cyclization in the presence of acidic conditions and other enzymes to yield *C-*glycosides. Our study revealed the existence of *AjCGT1*, a *C-*glycosyltransferase capable of catalyzing the biosynthesis of *C-*glycosylated GAs in plants, marking the first time such an enzyme has been identified. *AjCGT1* possesses a unique characteristic as it promotes C-2 glycosylation of 4-O-Me-GA. Under acidic conditions, the -OH group of glucose on 2-Glucosyl-4-O-Me-GA undergoes esterification dehydration with the -COOH group of 4-O-Me-GA, ultimately resulting in the production of bergenin. This is not only the first confirmed CGT involved in the biosynthesis of *C-*glycosylated GAs, but also the first demonstration that the -OH group of glucose participates in the formation of *C-*glycosides. Further, our findings serve as a valuable reference for future exploration of other types of CGTs.

In recent years, GA biosynthesis has been accomplished in microorganisms, primarily via modifications to the 3-DHS and chorismate pathways (Xinxiao et al., 2021). Introduction of 3-DHS dehydratase (AroZ) and 4-hydroxybenzoic acid ester hydroxylase (PobA Y385F) in engineered yeast under fed-batch conditions resulted in the production of 20 g/L GA (Kambourakis et al., 2000). GA production of 440.53 mg/L was achieved by introducing the more efficient double mutant Y385F/T294A, which continuously hydroxylates 4-HBA to GA (Chen et al., 2017). *S. cerevisiae* overexpressing upstream genes of the SA pathway and AroZ and PobA Y385F produced 682 mg/L GA (Brückner et al., 2018). The introduction of chorismate lyase (UbiC) effectively increased the flux of 4-HBA and promoted the synthesis of downstream products (Shang et al., 2020). Based on these findings, the UbiC and PobAY385F/T294A genes will be optimized and integrated with *BpSDH1*, *BpOMT1* or *AjOMT1*, and *AjCGT1* into engineered yeast. In conclusion, this study uncovered the catalytic functions of an SDH (*BpSDH1*), two OMTs (*BpOMT1* and *AjOMT1*), and a CGT (*AjCGT1*) involved in the bergenin biosynthetic pathway in *B. purpurascens* and *A. japonica*. The molecular mechanism of bergenin biosynthesis was elucidated for the first time, paving the way to *de novo* biosynthesis in engineered yeast.

## Data availability statement

The datasets presented in this study can be found in online repositories. The names of the repository/repositories and accession number(s) can be found below: NCBI accession numbers: PRJNA1008861 and PRJNA1008855.

## Author contributions

X-YL: Data curation, Formal analysis, Investigation, Methodology, Software, Validation, Visualization, Writing – original draft, Writing – review & editing. Y-NW: Data curation, Investigation, Validation, Writing – review & editing. J-SD: Data curation, Investigation, Writing – original draft. B-HC: Formal analysis, Supervision, Software, Writing – original draft. K-YL: Data curation, Supervision, Writing – review & editing. LF: Investigation, Methodology, Project administration, Writing – review & editing. G-SX: Conceptualization, Data curation, Formal analysis, Software, Writing – original draft. S-YZ: Formal analysis, Visualization, Writing – review & editing. Y-CL: Supervision, Writing – review & editing. S-CY: Funding acquisition, Supervision, Writing – review & editing. G-HZ: Funding acquisition, Resources, Supervision, Writing – original draft, Writing – review & editing. BH: Funding acquisition, Resources, Supervision, Writing – original draft, Writing – review & editing.

## Supplementary material

Supplementary Figure 1 SDS-PAGE analysis of protein expression for BpSDH2, BpOMT1, AjOMT1, BpCGT1, and AjCGT1. **(A–E)** respectively represent the SDS-PAGE analysis results of the protein expressions of BpSDH2, BpOMT1, AjOMT1, BpCGT1, and AjCGT1. M represented the Direct-load™ Color Prestained Protein Marker; 1 represented the electrophoretic results of SDS-PAGE protein in 250 mM imidazole flow penetrating solution.

Supplementary Figure 2 The extracted ion chromatogram (EICs) of compounds with molecular weights of 154, 170, and 172. **(A)** The extracted ion chromatogram (EICs) of the protease activity extract of BpSDH2 protein. **(B)** The extracted ion chromatogram (EICs) of standard PCA, 3-DHS, and GA.

Supplementary Figure 3 The identification of reaction products and standards by LC-MS/MS analysis. **(A, C, E)** represent characteristic ion peaks with molecular weights of 172, 170, and 154, respectively, in the enzyme activity extract of *BpSDH2*. **(B, D, F)** represent the characteristic ion peaks of the standard with molecular weights of 172 (3-DHS), 170 (GA), and 154 (PCA), respectively.

Supplementary Figure 4 The identification of reaction products and 4-*O*-Me-GA standard of OMT protein by LC-MS/MS analysis. **(A)** The extracted ion chromatogram (EICs) of the enzyme activity extracts of *BpOMT1* and *AJOMT1* with a molecular weight of 184, as well as the extracted ion chromatogram (EICs) of the standard 4-*O*-Me-GA. **(B)** The characteristic ion peak of the standard 4-*O*-Me-GA with a molecular weight of 184. **(C)** The characteristic ion peak with a molecular weight of 184 in the enzyme activity extract of *BpOMT1*. **(D)** The characteristic ion peak with a molecular weight of 184 in the enzyme activity extract of *AjOMT1*.

Supplementary Figure 5 The identification of products of CGT protein and bergenin standard by LC-MS/MS analysis. **(A)** The extracted ion chromatogram (EICs) of the enzyme activity extracts of *AJCGT1* with a molecular weight of 346 under conditions in the presence of methanol. **(B)** The MS fragments of the enzyme activity extracts of *AJCGT1* with a molecular weight of 346 under conditions in the presence of methanol. **(C)** The characteristic ion peak with a molecular weight of 346 in the enzyme activity extract of *AjCGT1* under conditions in the presence of methanol. **(D)** The extracted ion chromatogram (EICs) of the enzyme activity extracts of *AJCGT1* with a molecular weight of 328 under conditions in the presence of hydrochloric acid, as well as the extracted ion chromatogram (EICs) of the standard bergenin. **(E)** The characteristic ion peak of the standard bergenin with a molecular weight of 328. **(F)** The characteristic ion peak with a molecular weight of 328 in the enzyme activity extract of *AjCGT1* under conditions in the presence of hydrochloric acid.

Supplementary Figure 6 Alignment of amino acid sequences of shikimate dehydrogenase *BpSDH2*, *AjSDH2*, *VvSDH3*, *CsDQD/SDHc*, and *EcDQD/SDH3*. Amino acid sequences alignment were performed using DNAMAN 8.0.
