# Supplementary figures and images for: Biosynthetic pathway of prescription bergenin from Bergenia purpurascens and Ardisia japonica
Source: Front Plant Sci. 2024 Jan 4;14:1259347. doi: 10.3389/fpls.2023.1259347 (PMC10794647; doi:10.3389/fpls.2023.1259347)

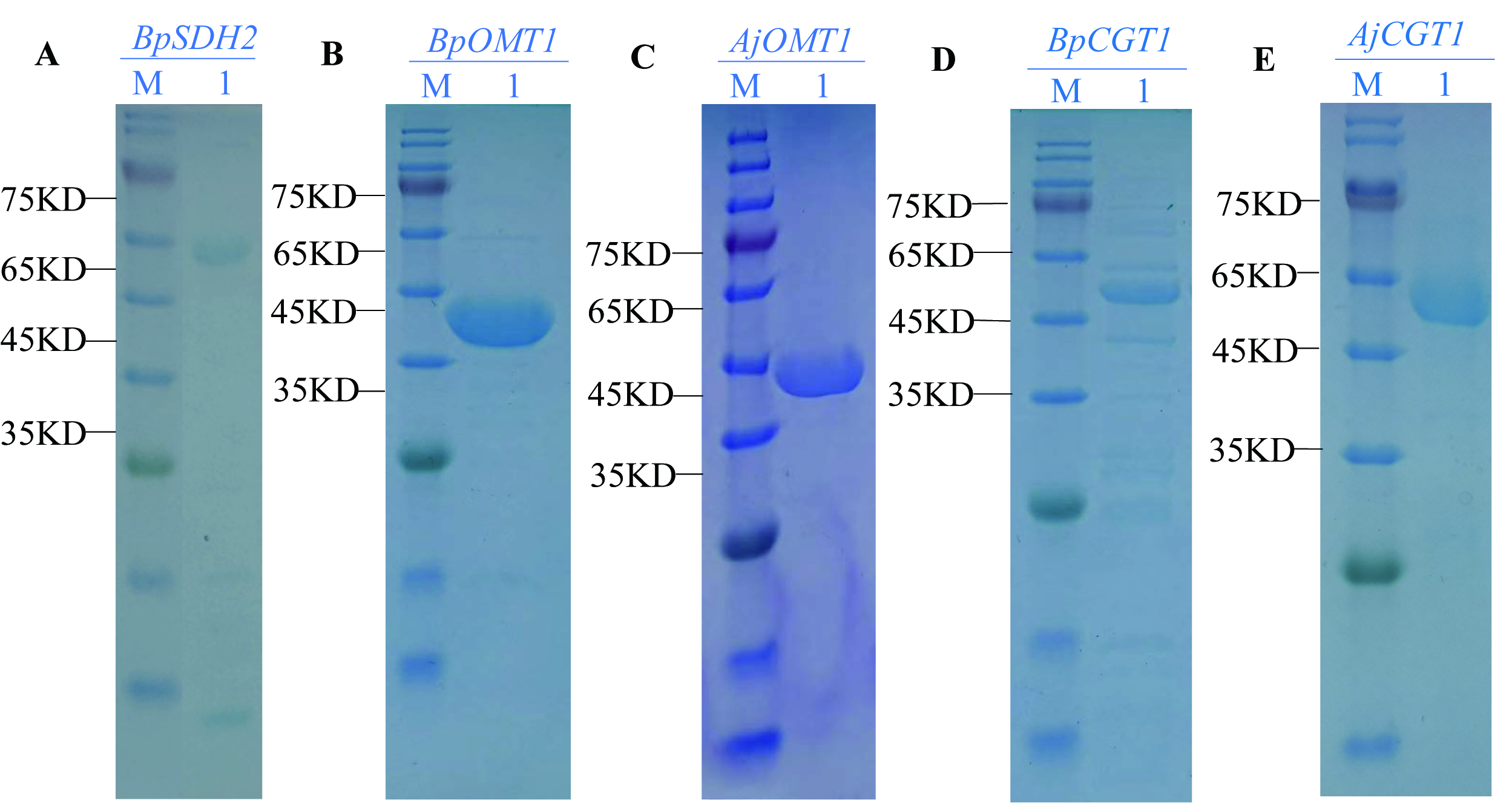

Supplement: Supplementary Figure 1 — SDS-PAGE analysis of protein expression for BpSDH2, BpOMT1, AjOMT1, BpCGT1, and AjCGT1. (A–E) respectively represent the SDS-PAGE analysis results of the protein expressions of BpSDH2, BpOMT1, AjOMT1, BpCGT1, and AjCGT1. M represented the Direct-load™ Color Prestained Protein Marker; 1 represented the electrophoretic results of SDS-PAGE protein in 250 mM imidazole flow penetrating solution. [file Image_1.jpeg]

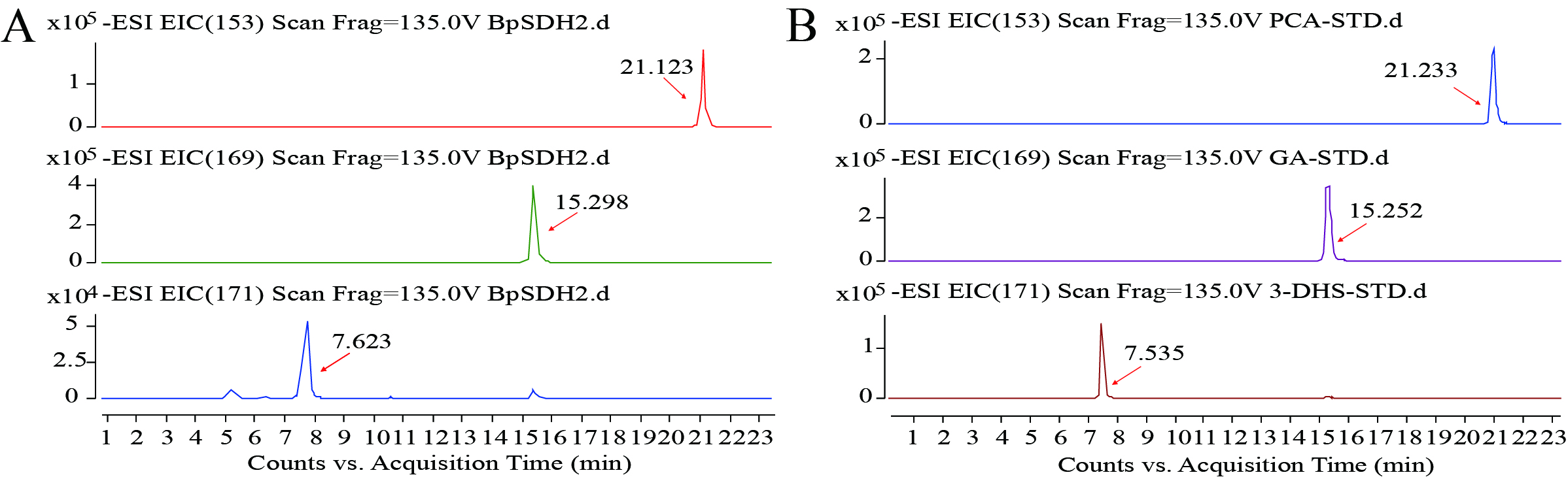

Supplement: Supplementary Figure 2 — The extracted ion chromatogram (EICs) of compounds with molecular weights of 154, 170, and 172. (A) The extracted ion chromatogram (EICs) of the protease activity extract of BpSDH2 protein. (B) The extracted ion chromatogram (EICs) of standard PCA, 3-DHS, and GA. [file Image_2.jpeg]

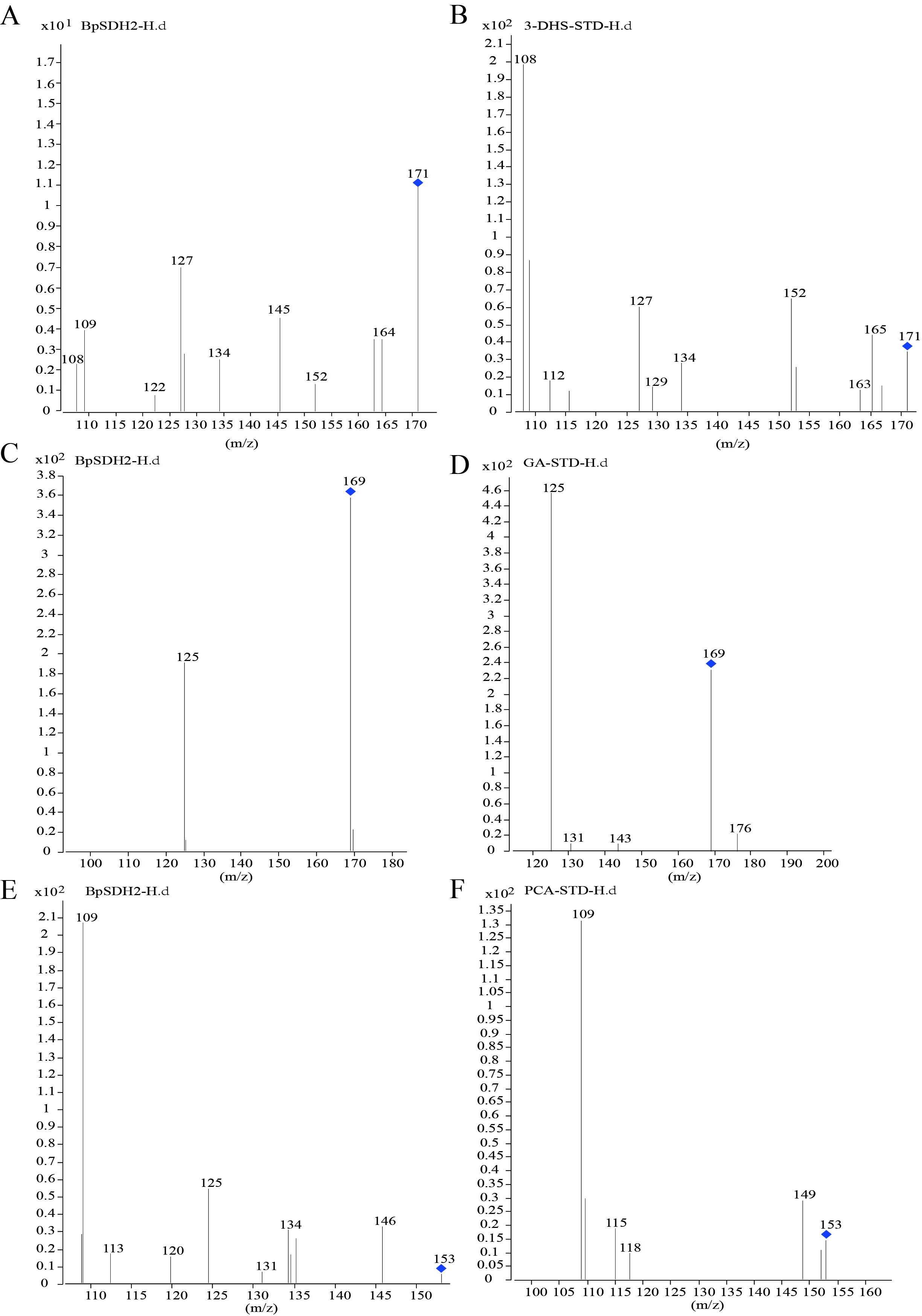

Supplement: Supplementary Figure 3 — The identification of reaction products and standards by LC-MS/MS analysis. (A, C, E) represent characteristic ion peaks with molecular weights of 172, 170, and 154, respectively, in the enzyme activity extract of BpSDH2. (B, D, F) represent the characteristic ion peaks of the standard with molecular weights of 172 (3-DHS), 170 (GA), and 154 (PCA), respectively. [file Image_3.jpeg]

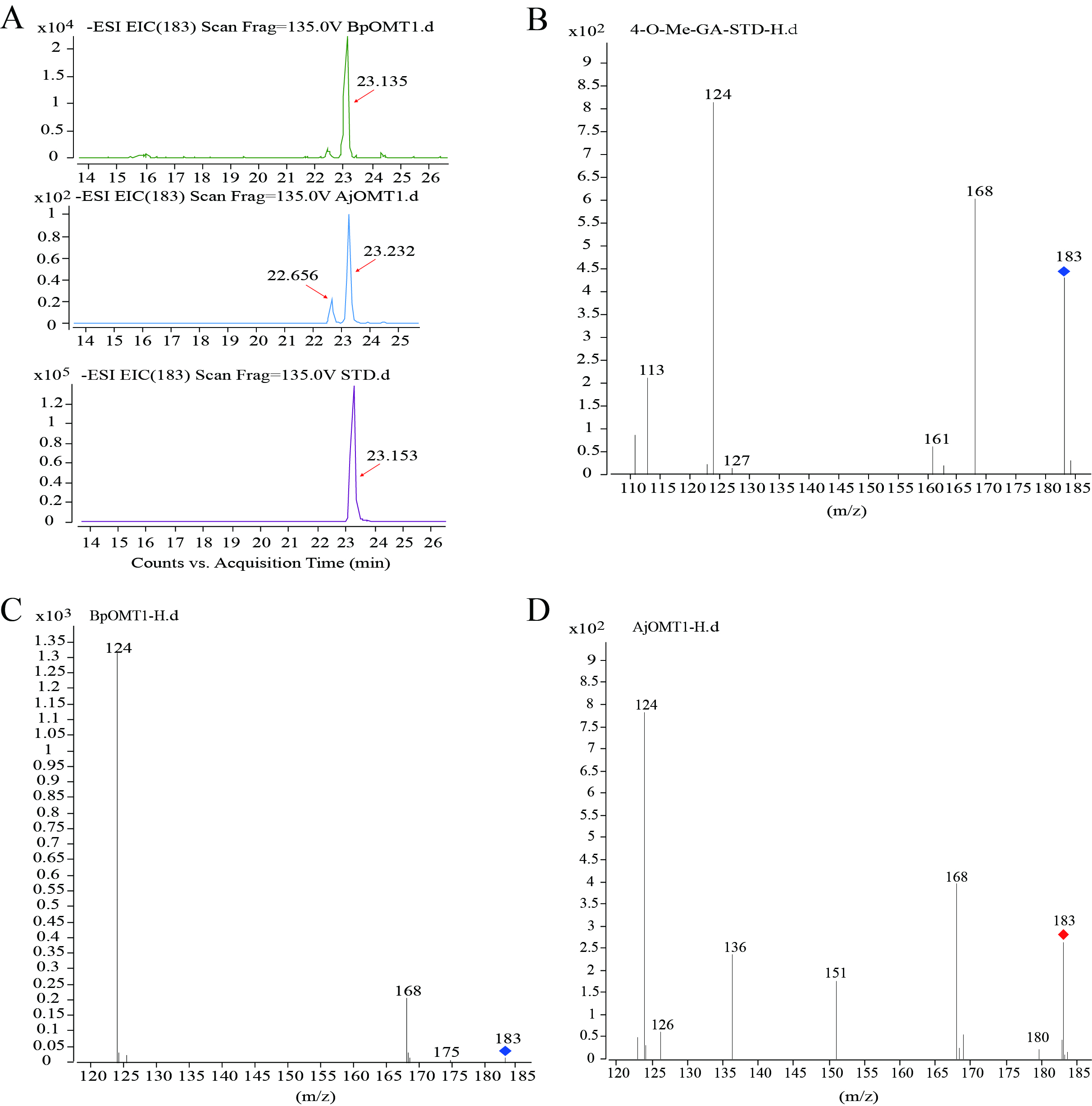

Supplement: Supplementary Figure 4 — The identification of reaction products and 4-O-Me-GA standard of OMT protein by LC-MS/MS analysis. (A) The extracted ion chromatogram (EICs) of the enzyme activity extracts of BpOMT1 and AJOMT1 with a molecular weight of 184, as well as the extracted ion chromatogram (EICs) of the standard 4-O-Me-GA. (B) The characteristic ion peak of the standard 4-O-Me-GA with a molecular weight of 184. (C) The characteristic ion peak with a molecular weight of 184 in the enzyme activity extract of BpOMT1. (D) The characteristic ion peak with a molecular weight of 184 in the enzyme activity extract of AjOMT1. [file Image_4.jpeg]

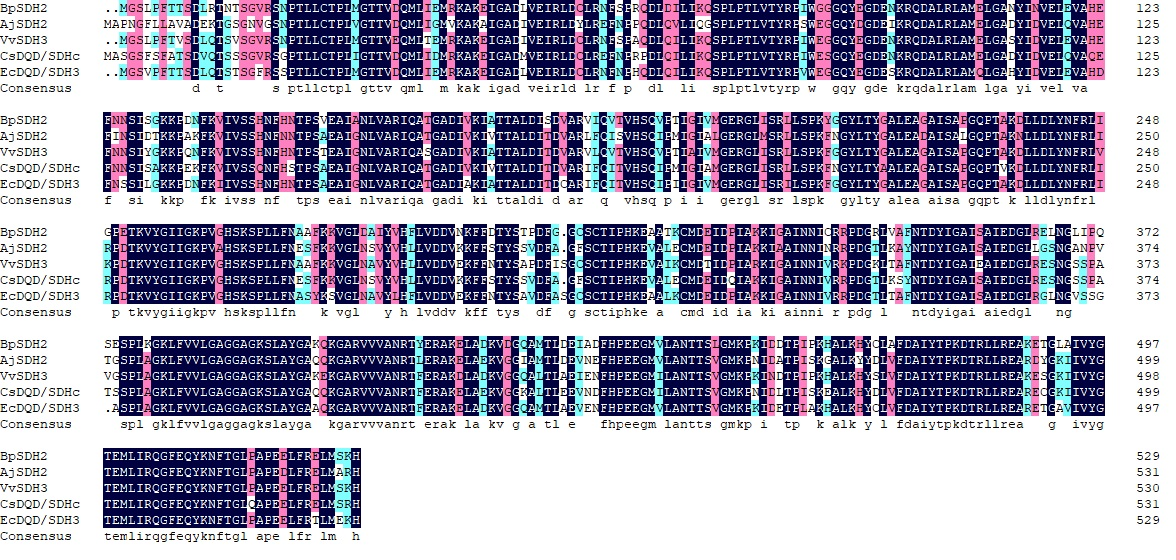

Supplement: Supplementary Figure 5 — The identification of products of CGT protein and bergenin standard by LC-MS/MS analysis. (A) The extracted ion chromatogram (EICs) of the enzyme activity extracts of AJCGT1 with a molecular weight of 346 under conditions in the presence of methanol. (B) The MS fragments of the enzyme activity extracts of AJCGT1 with a molecular weight of 346 under conditions in the presence of methanol. (C) The characteristic ion peak with a molecular weight of 346 in the enzyme activity extract of AjCGT1 under conditions in the presence of methanol. (D) The extracted ion chromatogram (EICs) of the enzyme activity extracts of AJCGT1 with a molecular weight of 328 under conditions in the presence of hydrochloric acid, as well as the extracted ion chromatogram (EICs) of the standard bergenin. (E) The characteristic ion peak of the standard bergenin with a molecular weight of 328. (F) The characteristic ion peak with a molecular weight of 328 in the enzyme activity extract of AjCGT1 under conditions in the presence of hydrochloric acid. [file Image_5.jpeg]

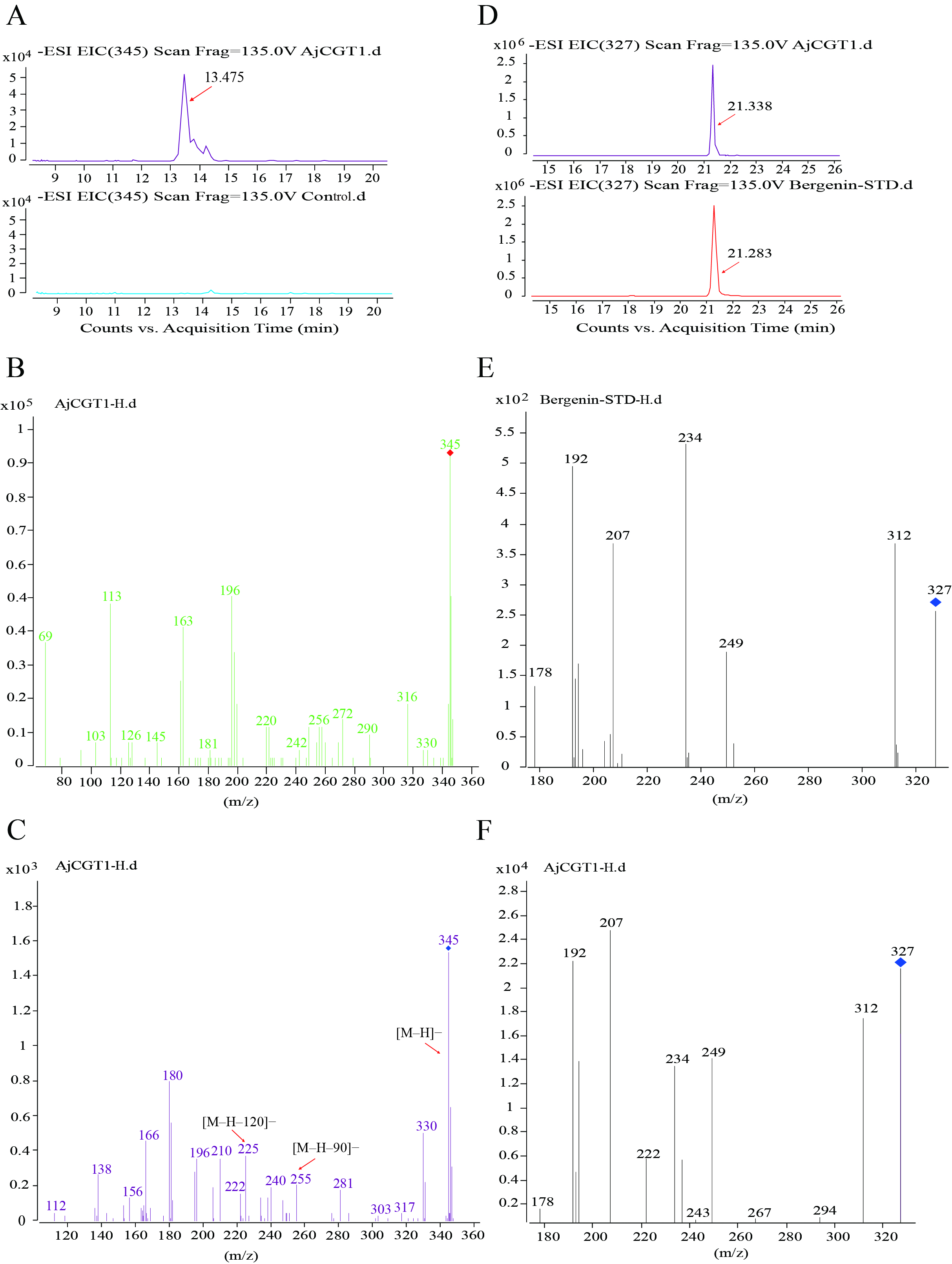

Supplement: Supplementary Figure 6 — Alignment of amino acid sequences of shikimate dehydrogenase BpSDH2, AjSDH2, VvSDH3, CsDQD/SDHc, and EcDQD/SDH3. Amino acid sequences alignment were performed using DNAMAN 8.0. [file Image_6.jpeg]
