# Supplementary material for: Biosynthetic pathway of prescription bergenin from Bergenia purpurascens and Ardisia japonica
Source: Front Plant Sci. 2024 Jan 4;14:1259347. doi: 10.3389/fpls.2023.1259347 (PMC10794647; doi:10.3389/fpls.2023.1259347)
Supplement: Supplementary file 7 [file DataSheet_1.docx]

>AjSDH2

ATGCAGTCGGTTGCTGCTGGTAGTGTGAAAATGGAGAGTTTAAGGAACAACAATCCGACCTTAATTTGTGCTCCAATAATGGGGGACTCAGTTGATCAGATGGTTATACATATGAATAAGGCTAAATCAAAGGGTGCTGATTTAGTAGAAATTCGATTGGATAGTTTGAAGAGCTTTAGCCCTTCTCAGGACCTTCAAACCCTAATCAAGCAGTGCCCTCTGCCTACCCTTTTCACTTACAGACCAAAATGGGAAGGCGGTCAATATGACGGTAATGAGAGCAAACGAGTGGAAACGCTTCGATTAGCTATGGAGTTGGGAGCTGATTACATTGATGTTGAGTTTCAGGTTGCTCATGAGTTTATTAGTGCCATGCATGGAACAAAGCCTGAAAAATGTAAAGTTATTGTTTCATCGCATAACTATGATTGTACTCCTTCTATTGAGGATCTTGGCGACCTTGTAGCGAGAATACAATCAACTGGAGCTGACATTGTGAAGTTCGCAACAACTGCCTTGGATATCACTGACGTGGCACGAGTTTTTCAAATTATCGTTCATTCTCAAGTCCCAATTATAGCACTCGTAATGGGTGAAAGGGGATTGATGTCCCGGATTCTTTGTCCCAAATTTGGAGGATATCTCACTTTTGGTACCCTTGAGGCCGGAGTAGTGTCTGCACCTGGGCAACCAACTATTGATGATCTGGTGAACTTGTACAATTTTAGACAGATTGGGCCTGATACAAAAGTGTTTGGCATCATTGGGAAGCCTGTAAGCCACAGCAAGTCACCGGCTTTGTACAACGAAGCATTCAAAGAAGTCGGTTTTAATGGGGTTTATCTTCATTTGTTGATCGATGATGTTGCAAAGTTCTTTGAGACTTATTCTTCTACAGATTTTGCCGGATTCAGTTGTACTATTCCTCACAAGGAGGCTGCAGTAAAGTGTTGTGATGAGGTTGATCCGGTTGCGGAGTCAATTGGAGCAGTTAATTGCGTCATCAGGAAACCAAGTGATGGGAAGTTATTTGGTTGTAATACAGACTATCTCGGTGCCATTTCCGCTATAGAGGATGCACTACGAGGCTCAAGTCATTCAAGTGGCACCACCGTGTCTCCATTGGCTGGTAAAGTTTTTGTGGTTATTGGTGCTGGTGGCGCGGGCAAGGCACTTGCTTATGGTGCAAAGGAAAAGGGTGCAAGAGTTGTGATAGCGAATCGCACCTATGAACGAGCTAAAGAACTTGCTGATACGATTGGAGGAGATGCTTTATCTCTTCAAGATCTAAATAATTTTCATCCAGAGGATGGTATGATCCTTGCAAACACGACTTCAATAGGCATGCAACCAAAAGTTGACGAGACACCTATTTCCAAGCAAGCTCTTAGATCATATGCACTGGTATTCGATGCTGTTTACACCCCCAAAATCACACAGCTGCTACGCGAAGCTGAAGAATCGGGAGCCAAAATTGTTTCTGGGGTGGAGATGTTCATTGGGCAGGCATATGAACAATTCGAGAAGTTCACTGGATTGCCTGCGCCTACGGAACTATTTAGGAAAATTATGGCAAAGTATTGA

>BpSDH2

ATGGGAAGCCTTCCGTTTACAACTTCTGATTTGCGAACGAATACAAGTGGAGTTAGGAGCAATCCAACTTTGCTATGCACGCCTTTAATGGGAACAACGGTGGATCAAATGCTGATTGAGATGAGAAAAGCTAAGGAAATTGGGGCCGACCTTGTTGAAATCAGGCTTGATTGTCTTAGAAATTTTAGTCCTCGCCAAGATCTTGATATCCTCATTAAGCAATCTCCTTTGCCTACTCTTGTCACTTACAGGCCAATATGGGGAGGTGGTCAATATGAAGGTGATGAAAACAAGAGACAGGATGCACTTAGGCTAGCTATGGAATTGGGAGCTAATTATATTAATGTTGAACTTGAGGTTGCTCATGAGTTCAACAATTCAATTTCTGGGAAAAAGCCTGATAATTTCAAAGTAATTGTGTCCTCTCACAATTTCCACAACACTCCATCTGTGGAGGCCATTGCCAATCTTGTGGCCAGAATTCAAGCTACTGGTGCTGATATAGTGAAAATTGCAACAACTGCATTGGACATCTCTGATGTTGCCCGCGTTATCCAAGTAACCGTGCATTCTCAAGTCCCAACAATTGGAATTGTAATGGGAGAGAGAGGCTTGATTTCGCGGTTACTTAGCCCTAAATATGGTGGATATCTCACTTATGGTGCACTTGAAGCCGGTGCAATATCAGCTCCTGGACAGCCAACTGCAAAAGATTTGTTAGATTTATACAACTTCAGACTTATAGGACCTGAAACCAAAGTCTATGGCATTATTGGGAAGCCTGTTGGACATAGCAAGAGTCCTCTTTTATTCAATGCAGCATTTAAAAAAGTCGGCCTTGACGCCATTTATGTGCATTTTTTGGTGGACGATGTCAACAAGTTTTTCGATACCTATTCGACCCCTGATTTTGGTGGATGCAGTTGCACAATTCCTCACAAGGAGGCTGCAACGAAATGCATGGACGAGATCGACCCAATTGCTAAGAAAATTGGAGCAATTAATAATATCTGTAGGAGACCTGATGGAAGATTAGTTGCTTTTAACACGGACTACATTGGCGCCATTTCTGCCATTGAAGATGGATTACGAGAATTGAATGGACTAATTCCTCAATCTGAATCACCCTTGAAGGGTAAGTTGTTTGTTGTTTTGGGAGCTGGTGGTGCTGGCAAGTCACTTGCTTATGGTGCAAAACAAAAGGGGGCAAGAGTTGTAGTTGCTAACCGAACCTACGAACGAGCTAAAGAGCTTGCGGACAAAGTTGATGGACAAGCTATGACTCTTGATGAAATAGCAGATTTCCATCCAGAAGAAGGGATGGTTCTTGCTAACACTACATCTTTAGGAATGAAACCTAAAATTGATGACACCCCCATTCCTAAGCATGCTCTGAAACATTATTGCTTGGCCTTTGATGCCATTTACACTCCGAAAGACACCAGGCTCTTGAGGGAAGCTAAAGAGACAGGACTTGCTATTGTTTATGGGACAGAAATGTTGATCCGCCAAGGCTTTGAACAATACAAGAACTTCACTGGTTTGCCAGCACCGGAGGAACTGTTTAGGGAACTTATGTCAAAACATGCTTAA

>BpOMT1

ATGGCTCCACAAAATGAAGCAGCAGCAGCTGAAGAAGAAGCAAACCTATTTGCCATGCAACTAGCAAGTTTATCAGTCTTACCAATGGTACTTAAATCAGCCATAGAACTAGACCTTCTAGAAATCATGGCTAAATCTGGCCCAGGTGCATACATGTCACCCATAGATATAGCGTCTCAGCTTCCTACAAACAATCCAGATGCACCTGTCATGCTCGACCGCATTTTGCGCCTGCTAGCATGCTACTCTGTTCTCACTTGCTCTGTCCGAAATCTCCCTGATGGCCGTGTTGAGAGGCTTTATGGTCTAGCACCTGTTTGTAAGTACTTGACCAAGAACGAGGATGGTGTCTCCATTGCTGCTCTTTGTCTCATGAATCAAGACAAGATCCTCATGGAGAGCTGGTACCACTTGAAAGATGCAGTTCTTGATGGTGGCATTCCATTCAACAAGGCTTATGGAATGTCTGCCTTCGAGTACCACGGCACGGATCCTAGATTCAACAAGGTTTTTAACAGGGGAATGTCTGATCACTCAACAATTACCATGAAGAAAATCCTTGAGACATACGAAGGATTTGAGGGACTCACATCTGTCGTCGACGTTGGTGGTGGTACTGGAGCCACTCTTAACATGATCCTCTCCAAGTATCCCAACATTAGGGGCATTAACTTTGATTTGCCTCATGTGATTGAGGATGCCCCATCTTATCCTGGTGTGGAGCATGTTGGAGGAGACATGTTTGTTAGTGTTCCAAAAGGGGATGCTATTTTCATGAAGTGGATATGTCATGACTGGAGCGACGAACACTGCTTGAAATTTTTGAAGAATTGCTATGATGCACTTCCGAGCAATGGGAAGGTGATTCTTGCTGAATGCATTCTTCCAGTAACGCCGGACACTAGCCTTGCAACTAAAGGAGTTATCCATATCGATGTGATCATGTTAGCGCATAATCCAGGAGGGAAGGAAAGGACTGAGAAGGAGTTTGAGGCCTTGGCAAAAGGTGCTGGATTTCAAGGCTTCCAAGTATTCTGCAATGCTTTCAATTCTTATATCATGGAATTCTTGAAGTAG

>AjOMT1

ATGGGTTCCTTAGAAAACACCCAAACAACCCAAAACGAGGAAGAACAAGCTTGCCTTTTCGCCATGCAACTGGCTAGCGCCTCTGTTCTCCCCATGATTTTGAAATCGGCGATCGAGCTCGACTTGCTCGAGATTATTGCGAAGGCGGGAGAGGGGGCATTTCTGTCCCCGAAGGAGATTGTGAAGGAGATTCCGACGAGAAACCCCGAGGCAGCGGTGATGCTTGATCGGATTCTAAGGTTGTTGGCGAGTTACTCTGTTTTGAAGTGTGTTGTGAGAGAAACGGAAGACGGGGCGCCCGAGAGGCTGTACGGGGTGGCCCCGGTGTGTAAGTACTTGACAAGGAACGAGGACGGGGTTTCGATGGCGGGTTTGCTGCTGATGAACCAGGATAAGGTGCTCATGGAGAGCTGGTATCATTTGAAAGATGCTGTGTTAGAGGGTGGGATACCCTTCAACAAGGCCTATGGAATGAGTGCATTTGAGTACCATGGCACTGATCAAAGATTCAACAAGGTGTTCAACAATGGAATGTCTAACCACTCCACAATTACAATGAAGAAAATACTCGAGGTCTACACAGGGTTCGAAGGGCTCGGTTCATTGGTCGATGTCGGTGGCGGGGTCGGGGCCACCCTAAACATGATTATCTCCAAGTACCCCTCCATTAAGGGCATCAATTTCGACTTGCCTCATGTTATCGAAGATGCCCCAGCTCTCCCAGGTGTGGAACATGTTGGAGGAGACATGTTTGTAAGCGTACCAAAGGGAGATGCCATTTTTATGAAGTGGATATGCCATGATTGGAGCGACGCCCACTGCTTGAAATTCTTGAAAAATTGCTATGACGCTCTACCCCAAAACGGGAAGGTGATCATCTGCGAGTGCGTTCTCCCTGCATACCCGGACACCAGTCTAGAGACCAAGAACGTGGTTCACGTTGATGTGATCATGTTGGCACACAACCCGGGTGGCAAAGAGAGGACCGAGAAAGAGTTTCAGGATTTGGCAAAGAGCGCTGGCTTTGCAGGGTTCCGAGTGATGTGCTCCGCCAACAATACGAGTGTCATGGAGTTTCTCAAGAGTAATGTTTAA

>AJCGT1

ATGTCTAACACCGGCAACCAAGAACCACGGCCGCACATCGCCCTCCTTCCCTCTGCCGGTATGGGCCACCTCAATCCGCTCCTCCGCCTTACCGCCACGCTGGCAGCCCGGGGCTGTCATGTCACTGTCATCACGCCCATACCGACATTCACTGCCGCAGAGTCCGAACACATGGACGAGTTTTTCTCCACCTACCCGAATATCAGCCGCCTCAAGTTCCAAATTGTTGGTGACGAGCCCCCCTCCGAGTTCGACCCGCCCATTTTCCTCCAGTGGCAGTCAATTAACCGCTCCGGCCACATCCTTAGGCCACTTCTAACTGCTCTCTCCCCGCCTATCACAGCCCTTTTCTCCGACTTTGTTGCGGGCCTCTGTATCTGCCCCATTGCCGACGATCTCAAAATACCAAATTACTTCGTTGTCACCTCTTCGGCTCGGTTCTTTTCCCTCTGGGCTCTCCTACCTTATATCAACAAAAGCCACGACGACGAAATACTAATCCCGGGCTTATCCCCATTTCACATATCTACTATCCCCCCACCATTTTTCAACCCAAATCATCTCTTCACTAAACTCGCAACCTCGAATGCTCCACTGTTGTCTAAGGCGAAAGGGATCTTGCTGAACACCTTTGACTATTTCGAACCCGATACAATCACTGCGATTAGAAAAGGTACCGTTCTAAGCGATCTGCCGCAAGTTTTCCCGATTGGCCCCTTGAAAGATTATAAATTTGGAAAAGGGAATTACCCCACGTGGCTAGACGATCAAGAGGAGGGATCCGTGGTGTATGTAAGCTTCGGAAGCAGAACCACACTGTCGAGGGATCAAATCAGGGAGCTTGGGGATGGGCTTGAAAGGAGCGGGTTTCCGTTTTTATGGGTCTTGAAAACGAGCAAAGAGGACGAAGGAGAGCTGCGAGATTTGTTGGGTGATACATTCTTCGAGAAAACGGGAAAGAAGGGGATTGTAGTGAAGGGGTGGGCCGATCAGGAAGCGATCCTGGCCCACCCAGCGATCGGGGGTTTCGTGAGCCACTGCGGGTGGAACTCCGTAACGGAGACTACTCGATTCGGGGTCCCGATCCTAGCTTGGCCACAACATGGGGATCAGCGGTTGAATGCGGAGGTGGTGGAGAAGGCCGGAATGGGGGTTTGGGAGAGAGATTGGGGGTGGCAGAGAGAGAGATTGATAAAGGGGGAGGAGATTGGGGAGAAGATTGAGAAGTGGATGACTGATGAGAAGTCGAAGACTCAAGCTAAGAAGATTAGAGTTGAAGCTAAAAAAGCTCTCGAGGCTAATGGGAGCTCTGAGAAGGCTTTATCTGATTTAATTACTACGGTGAAGAAAAATTAG

>BpCGT1

ATGTTTCGCGCCAAGGCCGAAAGATACCAGCTCGCAAGGTTTTTTTTATGGATAGGGTTTAATATCTCTGATTTCCAAAGATCTCCTCACATAGCTCTCTTCCCAAGTGCTGGAATGGGTCATCTGACCCCCTTCCTTCGGCTTGCTGCTATGCTGTCTTCCCGGAACTGTTTGATCACTCTCATCACTGCCAAACCAACTGTGTCAGCCGCAGAATCCACTCATATTTCTTCCTTCATTTCAACCCACCCAGAAATCAGGCATCTTGAGTTCCAAATACTTCCCATTAACGCCACCAATCCCACCACTGATGATCCCTTCTTTATCCAATTCGAAGCAGTTAGCCGATCTGTTCATGTGCTCCATCCATTGCTATCTTCGGCATCTCCTCCATTGTGTGCTATTTTTTCTGATCTAGTTGTAGCTTCAAGTACTGCTCCAATAGCCGATGATCTCTGCATCCCGAACTACATCGTCTCCACCACCTCAGCCAAGTTTCTATGTCTCATAGCATACCTTCCAGTCCTAACATCCGACTCTGCTAAATTTAATCTCGGTTGTACTGAGGTCCAGATCCCAGGCCTTAGTCCACTGCCCATAACAAGTATTCCTCCACCATTCTGGAACACAAATCATCTCTTTACAGCACACTTGATTACAAATGCCCGAGCTCTTCCTAAAGCCAAAGGAATCATAATGAATACCTTCAATTGGTTTGAACAAGAGACGATTGATGCTGTCAGCAATGGCGGAGTCCTAGACAGTCTCCCACCCATTCTGCCAATTGGACCACTGAAACCATTTAGTTTTGAAAAGGACCAGAATCAGTATCTGTCATGGCTAGACAACCAACCAGCAGAATCTGTGGTGTACGTGAGCTTCGGAAGCAGAACAGCCATGTCAGAAGATCAAATAAGGGAATTGGGAATTGGGTTGGAGAGAAGCAAGCATAGATTCCTCTGGGTACTGAAGTCAAGTAAAGTAGATAAAGAAGATAGCAAAGACCTGAAAGAGTTGCTGGGAAATTTCTTTTTGGAAAGAACGAGCAACCAAGGGATAATAGTTAAGAGGTGGGTAAACCAGGAGGAGATTCTAGCACACCCTGCCATTGGAGGGTTCATTAATCATTGCGGCTGGAACTCGGTATCTGAAGCAGCATGGCAAGGGATACCCATATTGGCATGGCCTCAACATGGAGACCAGAGAGTTAACGCAGGCGTGGTGGAGAAAGTAGGGTTGGGGATATGGGAGAGGGGTTGGGGTTGGGGTGGTGAGAAGCTGGTGAAGGGTGTAGAGATTGGAGAGAGAATTGGGCAGTTGATGGAAGATGAGAAGCTGAGGAGTAGAGCCAGGAAGGTAGGAGAAGAGGCTAAGAAAGCGTGTGGTTTTGGCGGGAGCTCAGAAAAGGCGTTGACAGGAATAATCGATATGGTGACAAGGAACTAA
