# Supplementary material for: Biosynthetic pathway of prescription bergenin from Bergenia purpurascens and Ardisia japonica
Source: Front Plant Sci. 2024 Jan 4;14:1259347. doi: 10.3389/fpls.2023.1259347 (PMC10794647; doi:10.3389/fpls.2023.1259347)
Supplement: Supplementary file 8 [file Table_1.docx]

**Table S1.** Annotation Abstracts of Unigenes sequences of Transcriptome of *B. purpurascens* and *A. japonica*

| **Database** | ***B. purpurascens*** | | ***A. japonica*** | |
| --- | --- | --- | --- | --- |
|  | **Number of Annotation** | **Percentage** | **Number of Annotation** | **Percentage** |
| KEGG | 17,884 | 17.37% | 31,449 | 40.93% |
| KOG | 27,171 | 26.39% | 22,642 | 29.47% |
| SwissProt | 30,649 | 29.76% | 25,854 | 33.65% |
| Nr | 43,992 | 42.72% | 35,964 | 46.80% |
| Total | 44,726 | 43.43% | 37,356 | 48.61% |
