# Supplementary material for: Biosynthetic pathway of prescription bergenin from Bergenia purpurascens and Ardisia japonica
Source: Front Plant Sci. 2024 Jan 4;14:1259347. doi: 10.3389/fpls.2023.1259347 (PMC10794647; doi:10.3389/fpls.2023.1259347)
Supplement: Supplementary file 9 [file Table_2.docx]

**Table S2** Primers for recombination of candidate genes

| **Gene ID** | **primers with homology arms** |
| --- | --- |
| *AjSDH2* | F: gtggacagcaaatgggtcgcggatccATGCAGTCGGTTGCTG |
|  | R: tgtcgacggagctcgaattcggatccTCAATACTTTGCCATAATTTTCC |
| *BpSDH2* | F: gtggacagcaaatgggtcgcggatccATGGGAAGCCTTCCGTTTA |
|  | R: tgtcgacggagctcgaattcggatccTTAAGCATGTTTTGACATAAGTTCC |
| *BpOMT1* | F: gtggacagcaaatgggtcgcggatccATGGCTCCACAAAATGAAGCAG |
|  | R: tgtcgacggagctcgaattcggatccCTAATTTTTCTTCACCGTAGTAATTAAATC |
| *AjOMT1* | F: gtggacagcaaatgggtcgcggatccATGGGTTCCTTAGAAAACACCC |
|  | R: tgtcgacggagctcgaattcggatccTTAAACATTACTCTTGAGAAAC |
| *AjCGT1* | F: gtggacagcaaatgggtcgcggatccATGTCTAACACCGGCAACC |
|  | R: tgtcgacggagctcgaattcggatccCTAATTTTTCTTCACCGTAGTAATTAAATC |
| *BpCGT1* | F: gtggacagcaaatgggtcgcggatccATGTTTCGCGCCAAGGC |
|  | R: tgtcgacggagctcgaattcggatccTTAGTTCCTTGTCACCATATCGATTATTCC |
