# Supplementary material for: Biosynthetic pathway of prescription bergenin from Bergenia purpurascens and Ardisia japonica
Source: Front Plant Sci. 2024 Jan 4;14:1259347. doi: 10.3389/fpls.2023.1259347 (PMC10794647; doi:10.3389/fpls.2023.1259347)
Supplement: Supplementary file 10 [file Table_3.docx]

**Table S3** The ORFs of potential SDH/OMT/CGT genes discovered from other species.

| **No.** | **Gene** | **GenBank** | **Species** |
| --- | --- | --- | --- |
| 1 | *EcDQD/SDH1* | LC487988 | *Eucalyptus camaldulensis* |
| 2 | *EcDQD/SDH2* | LC487989 | *Eucalyptus camaldulensis* |
| 3 | *EcDQD/SDH3* | LC487990 | *Eucalyptus camaldulensis* |
| 4 | *EcDQD/SDH4a* | LC487991 | *Eucalyptus camaldulensis* |
| 5 | *EcDQD/SDH4b* | LC487992 | *Eucalyptus camaldulensis* |
| 6 | *VvSDH1* | KU163040 | *Vitis vinifera* |
| 7 | *VvSDH2* | KU163041 | *Vitis vinifera* |
| 8 | *VvSDH3* | KU163042 | *Vitis vinifera* |
| 9 | *VvSDH4* | KU163043 | *Vitis vinifera* |
| 10 | *CsDQD/SDHa* | MH000201 | *Camellia sinensis* |
| 11 | *CsDQD/SDHb* | MH000202 | *Camellia sinensis* |
| 12 | *CsDQD/SDHc* | MH000203 | *Camellia sinensis* |
| 13 | *CsDQD/SDHd* | MH000204 | *Camellia sinensis* |
| 14 | *aroE* | NP_417740.1 | *Escherichia coli* |
| 15 | *EjOMT1* | LC127201 | *Eriobotrya japonica* |
| 16* | *SlGOMT1* | - | *Silene latifolia* |
| 17* | *SlGOMT2* | - | *Silene latifolia* |
| 18 | *GFLOMT1* | KP176693 | *Papaver somniferum* |
| 19 | *GFLOMT2* | KP176694 | *Papaver somniferum* |
| 20 | *GFLOMT6* | KP176698 | *Papaver somniferum* |
| 21 | *CTOMT1* | NP_001306101 | *Solanum lycopersicum* |
| 22 | *ODOMT* | KHJ97991.1 | *Oesophagostomum dentatum* |
| 23 | *OsCGT* | CAQ77160 | *Oryza sativa* spp. indica |
| 24 | *UGT708A6* | NP_001132650.1 | *Zea mays* |
| 25 | *MiCGTb* | ALD83754.1 | *Mangifera indica* |
| 26 | *MiCGT* | AMM73095 | *Mangifera indica* |
| 27 | *FeCGTa* | BAP90360 | *Fagopyrum esculentum* |
| 28 | *FeCGTb* | BAP90361 | *Fagopyrum esculentum* |
| 29 | *UGT708D1* | BAR73279 | *Glycine max* |
| 30 | *FcCGT* | BBA18062 | *Fortunella crassifolia* |
| 31 | *CuCGT* | BBA18063 | *Citrus unshiu* |
| 32 | *GgCGT* | MH998596 | *Glycyrrhiza glabra* |
| 33 | *DcaCGT* | QOD39011.1 | *Dendrobium catenatum* |
| 34 | *AbCGT* | MN747045 | *Aloe barbadensis* |
| 35 | *NnCGT1* | XP_010258947.1 | *Nelumbo nucifera* |
| 36 | *NnCGT2* | XP_010265663.2 | *Nelumbo nucifera* |
| 37 | *SbCGTa* | MK894443 | *Scutellaria baicalensis* |
| 38 | *SbCGTb* | MK894444 | *Scutellaria baicalensis* |
| 39 | *DcUGT2* | ATL15304.1 | *Dactylopius coccus* |
| 40 | *gilGT* | AAP69578.2 | *Streptomyces griseoflavus* |
| 41 | *UrdGT2* | AAF00209 | *Streptomyces fradiae* |
| 42 | *IroB* | CAE55724 | *Escherichia coli* Nissle 1917 |
| 43 | *SsfS6* | ADE34512 | *Streptomyces* sp. SF2575 |

*, These genes were discovered in *Silene latifolia*. However, GenBank Accession numbers were not provided, and the protein sequences were obtained from Gupta et al., 2012.

**References:**

Gupta, A. K., Akhtar, T. A., Widmer, A., Pichersky, E., and Schiestl, F. P. (2012). Identification of white campion (silene latifolia) guaiacol o-methyltransferase involved in the biosynthesis of veratrole, a key volatile for pollinator attraction. *BMC Plant Biology*. 12, 158. doi:10.1186/1471-2229-12-158
